# Spatiotemporal epidemiology, geographic hotspots, and risk factor associations of drug-resistant tuberculosis incidence in Indonesia: a Bayesian hierarchical modelling approach

**DOI:** 10.1186/s40249-026-01418-9

**Published:** 2026-02-13

**Authors:** Abdillah Farkhan, Saranath Lawpoolsri, Ngamphol Soonthornworasiri, Tiffany Tiara Pakasi, Sulistyo Sulistyo, Alya Salsabila, Richard J. Maude, Henry Surendra, Chawarat Rotejanaprasert

**Affiliations:** 1 Department of Tropical Hygiene, Faculty of Tropical Medicine, Mahidol University, Bangkok, Thailand; 2 Office of Health Quarantine I Tarakan, Directorate General of Disease Control, Ministry of Health, Tarakan, Indonesia; 3 National Tuberculosis Program, Directorate General of Disease Control, Ministry of Health, Jakarta, Indonesia; 4 Centre for Tropical Medicine and Global Health, Nuffield Department of Medicine, University of Oxford, Oxford, UK; 5 The Open University, Milton Keynes, UK; 6 Mahidol-Oxford Tropical Medicine Research Unit, Faculty of Tropical Medicine, Mahidol University, Bangkok, Thailand; 7 Monash University Indonesia, Tangerang Selatan, Indonesia; 8 Oxford University Clinical Research Unit Indonesia, Jakarta, Indonesia

**Keywords:** Spatiotemporal, Drug-resistant tuberculosis, Indonesia, Bayesian

## Abstract

**Background:**

Indonesia ranks among the countries with the highest burden of drug-resistant tuberculosis (DR-TB), contributing approximately 7.4% of global cases, many of which are likely underdiagnosed. To support targeted public health surveillance and control efforts, this study aimed to characterize the spatiotemporal distribution of DR-TB incidence in Indonesia, identify geographic hotspots, and examine associations with health system and socioeconomic factors.

**Methods:**

We conducted a nationwide retrospective analysis using annual DR-TB notification data from 2017 to 2022 across all 514 districts, obtained from the national tuberculosis information system. Multivariable Bayesian spatiotemporal regression models were fitted under alternative likelihood assumptions and space-time random effect structures. Model selection criteria were used to identify the best-fitting models for hotspot detection and estimation of risk factor associations.

**Results:**

DR-TB predominantly affected individuals aged 25–54 years, aligning with the working-age population. Hotspots were concentrated in urbanized regions, including the Jabodetabek megacity, Greater Surabaya, and districts in South Sumatra. The best-fitting model identified a protective association between first-line treatment success rates and DR-TB incidence [incidence rate ratio (IRR): 0.508; 95% credible interval (CrI): 0.368–0.702]. In contrast, DR-TB incidence was positively associated with the proportion of the population living below the poverty line (IRR: 1.028; 95% CrI: 1.013–1.044), households with improved sanitation access (IRR: 1.006; 95% CrI: 1.002–1.010), and increased municipal human development index (IRR: 1.068; 95% CrI: 1.049–1.094).

**Conclusions:**

DR-TB hotspots were primarily concentrated in urban areas, highlighting the need for targeted interventions. Improving first-line tuberculosis treatment success rates and addressing socioeconomic drivers, such as poverty, are critical for controlling DR-TB. Public health policies should prioritize workplace-based support for improving treatment adherence, provide safeguards for TB patients affected by poverty, and underscore the importance of a multisectoral TB surveillance and control program.

**Graphical Abstract:**

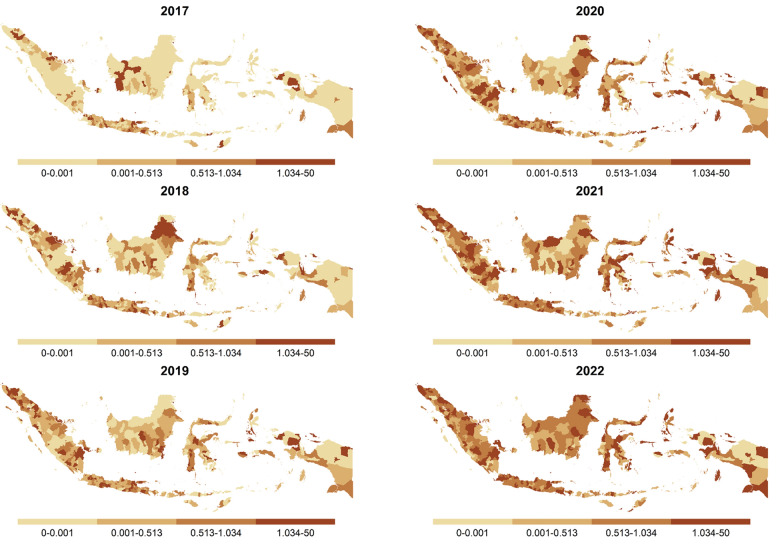

**Supplementary Information:**

The online version contains supplementary material available at 10.1186/s40249-026-01418-9.

## Background

Drug-resistant tuberculosis (DR-TB) remains a public health threat [1], affecting 3.2% of new cases and 16% of previously treated cases worldwide in 2023. Indonesia accounted for 7.4% of global DR-TB cases in 2023 ranking third worldwide [2]. The Indonesian Ministry of Health reported that the case detection rate for DR-TB in 2023 was only 40% of the 30,000 estimated cases, significantly below the national target of 80% [3]. Despite Indonesia aims to eliminate tuberculosis (TB) by 2030 [4]; however, half of the provinces still have first-line treatment coverage and treatment success rates below national targets, highlighting suboptimal efforts to prevent DR-TB among previously treated cases [3].

Several key factors contributing to DR-TB prevalence include treatment non-adherence, poverty, and inadequate healthcare resources [5]. Non-compliance during therapy for drug-susceptible tuberculosis (DS-TB) is recognized as the primary driver of disease progression [6–10], with previously treated TB patients being more likely to develop DR-TB strains [11]. TB is further linking to poverty, as the prolonged course of anti-TB therapy can impose financial strain on economically disadvantaged groups [12]. Although the national health insurance program in Indonesia provides financial coverage to ensure free TB treatment, access to healthcare is critical for accurate diagnosis, improving treatment outcomes, preventing DR-TB, and minimizing indirect out-of-pocket costs [13]. Evaluating these key contributing factors can help to comprehend the complex dynamics of DR-TB.

Spatial epidemiology plays a critical role in understanding the distribution of health events, identifying patterns, and linking them to risk factors, which is essential for guiding allocation of resources to high-risk areas [14, 15]. In TB research, frequentist-based spatial analysis methods have been used to identify regions with elevated TB risk through clustering techniques [16], to provide valuable insights into the determinants of TB using local geographically weighted regression (GWR) models [17, 18] and using spatial lag and spatial error models [19, 20]. Despite these advancements, frequentist methods may be inadequate for accurately estimating disease risk and identifying hotspots [21].

In contrast to frequentist approaches, Bayesian hierarchical spatiotemporal modelling can offer a more robust approach [22]. By incorporating spatiotemporal structures a priori, the Bayesian framework captures complex interactions between spatial and temporal factors, facilitating a more comprehensive analysis of disease spread. This method not only incorporates uncertainties but also provides more precise estimates of disease risk and hotspots [22, 23], enabling a deeper understanding of the spatial and temporal variation in disease incidence and its determinants [22, 24].

Research on drug-resistant tuberculosis incidence and its associated risk factors in Indonesia remains limited to specific provinces [25] or cities [26]. This study is one of the first nationwide studies that investigates the spatiotemporal distribution of DR-TB across all 514 districts in Indonesia, identifying geographical hotspots and modelling the associations between DR-TB incidence and potential risk factors, including TB treatment outcomes, health system variables, and socioeconomic conditions. Employing a Bayesian approach, it can provide district-level evidence to inform targeted public health policies and strategies in high-risk areas to improve treatment outcomes and address socioeconomic factors contributing to TB resistance, supporting TB control activities across the country.

## Methods

### Overview of DR-TB surveillance in the study site

Indonesia was divided into 38 provinces, each of which was further subdivided into districts, known locally as *kabupaten* (rural or semi-rural areas) and *kota* (urban municipalities), totalling 514 districts nationwide. In the country’s decentralized health system, districts functioned as the primary administrative units for health governance and service delivery, and therefore served as the foundation for disease control programs implementation. Reports on DR-TB cases were submitted by primary health centres, government hospitals, and private healthcare facilities through the national tuberculosis information system [*Sistem Informasi Tuberculosis* (SITB)]. The SITB functioned as a platform for healthcare providers to notify TB cases to their respective district health offices. During 2017–2022, SITB recorded a total of 3,146,975 notified TB cases, of which 54,291 were classified as DR-TB, representing 1.7% of all TB cases reported during this period. Among the 54,291, only 30,528 DR-TB cases received treatment, resulting in an enrolment rate of 56.2% for DR-TB during the period. Treatment coverage for TB in Indonesia has fluctuated in recent years, with a notable drop to 47% in 2020, before increasing again to 74.7% in 2022. Meanwhile, the treatment success rate has remained relatively stable, ranging from 83.1% in 2020 to 86.5% in 2022.

DR-TB was defined as tuberculosis caused by *Mycobacterium tuberculosis* complex bacteria that are resistant to at least one first-line anti-TB drug. The latest algorithm mentioned that DR-TB in Indonesia was diagnosed using the GeneXpert MTB/RIF rapid molecular test to detect resistant to rifampicin from all types of presumptive TB. The test was taken at designated primary healthcare centers, public hospitals, national laboratories for disease prevention, and public lung health centers. For patients whose GeneXpert MTB/RIF results indicate rifampicin resistance, further testing using a second-line Line Probe Assay (LPA) was conducted to detect resistance to fluoroquinolones and second-line injectable drugs. At a minimum, drug susceptibility testing should include rifampicin and isoniazid resistance screening for newly diagnosed patients, while patients with a history of previous TB treatment should undergo testing for fluoroquinolone resistance. [27, 28].

### Data design and data collection

We conducted a nationwide spatiotemporal analysis using aggregated secondary data from Indonesia's national tuberculosis program. District-level data on DR-TB notifications were collected from SITB that covers throughout 514 districts for the period 2017–2022. While this study focused on DR-TB cases identified through first-line TB drug testing, the cases also included individuals with additional resistance to second-line drugs, as indicated by the diagnostic algorithm described in the previous section. Ethical approval of this study was obtained from the Ethical Committee of the Faculty of Tropical Medicine, Mahidol University, Approval No. MUTM 2024-009-01. Since the data were de-identified, the requirement for informed consent was exempted.

DR-TB represents an advanced and complex form of TB that is shaped by treatment performance, health system capacity, and broader socioeconomic conditions that influence access to timely diagnosis and effective care [29, 30]. In this study, district-level potential risk factors were selected based on their epidemiological relevance reported in previous studies and their conceptual linkage to DR-TB pathways. Only variables with consistent nationwide availability throughout the study period were included to ensure comparability across districts. Detailed definitions and data sources for DR-TB indicators and associated risk factors are provided in supplementary document S1.

From SITB, we also obtained potential covariates related to first-line TB treatment outcomes, including treatment coverage, treatment completion rate, and treatment success rate. Treatment coverage was defined as proportion of TB patients who start the treatment among all TB cases. The treatment completion rate, which measured the proportion of patients who complete their treatment, was crucial for understanding adherence. It was important to note that patients who completed treatment but were not bacteriologically cured—due to insufficient evidence to confirm cure or a lack of clinical and microbiological response—typically required further evaluation and a switch to an alternative treatment regimen. The treatment success rate, defined as the proportion of cured patients among those treated, provided insight into the overall success of TB management in a district.

Health system-related indicators were also considered, reflecting the accessibility and effectiveness of TB care in each district. We obtained data on the number of health centers per 100,000 population from the National Statistical Agency. These health centers included primary health care facilities, public hospitals, and public lung health centers under the National Tuberculosis Program, and their distribution was critical in understanding access to timely TB diagnosis and treatment. More health centers indicated better availability of TB services, which may reduce barriers to care. Universal Health Coverage (UHC) data, sourced from the National Social Security Agency, was included to measure the proportion of the population covered by the national health insurance scheme, as this impacted access to TB care and influenced treatment coverage and adherence.

Socioeconomic factors were sourced from the National Statistical Agency, selected to capture the influence of socioeconomic conditions, as poverty and poor sanitation were well-established risk factors for TB. Data on household poverty described the percentage of the population with per capita expenditure below the poverty line. Meanwhile, household sanitation explained percentage of households which had good access to sanitation and were using improved sanitation facilities. Additionally, MHDI was included to reflect the overall development level of each district, as areas with lower human development often face greater challenges in healthcare access and TB management. By including these variables, the study aimed to identify a comprehensive set of factors influencing DR-TB incidence, which was crucial for designing more inclusive and effective public health interventions. This study followed Strengthening The Reporting of Observational Studies in Epidemiology (STROBE) reporting guidelines and the completed checklist was provided in Supplementary document S2 [31].

### Standardized incidence rate

Standardized incidence rates (SIRs) were used to compare DR-TB incidence across districts with differing population sizes and risk structures. Given the small number of DR-TB cases in many districts and the lack of complete age- and sex-specific notification data at the district level, indirect standardization was adopted. This approach yields more stable estimates for small-area disease mapping and is widely used in spatial epidemiology and Bayesian disease modelling [32, 33]. To calculate the SIR, we derived an indirect standardization by dividing the notified DR-TB cases, ${y}_{it}$ over those expected, ${E}_{it},$ given to this expression:

1 $$SI{R}_{it}= \frac{{y}_{it}}{{E}_{it}}$$

2 $${E}_{it}= \frac{{\sum }_{t=1}^{T}{y}_{it}}{{\sum }_{t=1}^{T}{N}_{it}}*{N}_{it}$$

where *i* was each administrative district for *t* year, (*i* = 1, 2…, 514); *t* = 1…,6). We employed all TB cases as the reference population or ${N}_{it}$. Then the expected cases ${E}_{it}$ was calculated as the national proportion of notified DR-TB multiplied by all notified TB cases in district *i* and *t* year. This meant that we standardized the expected cases according to the all-notified TB to interpret how likely DR-TB was among the population of all notified TB cases, considering that those infected with TB were at higher risk of developing or transmitting DR-TB. While DR-TB could also arise from direct transmission, focusing on previously-treated cases directly reflected the emergence of resistance due to inadequate or incomplete therapy. This approach enabled the control program to design and implement the targeted intervention to prevent the progression of DR-TB among TB populations. Using first-line outcomes allowed us to capture early indicators of resistance development before second-line treatment was initiated, facilitating timely intervention.

### Spatiotemporal Bayesian modelling for district DR-TB incidence in Indonesia

Bayesian disease mapping explored spatial and temporal relationships, highlighting how neighbouring areas and time periods influence disease patterns. The district-level DR-TB was modelled within a Bayesian inference framework [34–36]. We assumed the district DR-TB notifications ${y}_{it}$ to follow a Poisson distribution with a mean ${\upmu }_{i,t}$ and offset ${E}_{it}$:

3 $${y}_{it}{\sim Poisson (\upmu }_{it}, {E}_{it})$$

4 $$log \left({\upmu }_{it}\right){= log(E}_{it})+ {\theta }_{it}$$

5 $${\theta }_{it}= \upbeta{0}+{\sum }_{j = 1}^{J}\upbeta{\mathrm{j}}\text{ X}{\mathrm{it}}{\mathrm{j}}+ {u}_{i}+{v}_{i}+{T}_{t}+{\delta }_{it}$$

Here, ${\theta }_{it}$ was the linear predictor comprising the overall intercept $\upbeta{0}$, a set of *j* spatiotemporal risk factors ${\mathrm{X}}{\mathrm{itj}}$ with coefficients $\upbeta{\mathrm{j}}$, spatial random effects, ${u}_{i}$ and ${v}_{i}$, temporal random effects ${T}_{t}$, and spatiotemporal interaction terms ${\delta }_{it}$. The offset ${E}_{it}$ adjusted for population differences between districts.

**Fig. 1:**
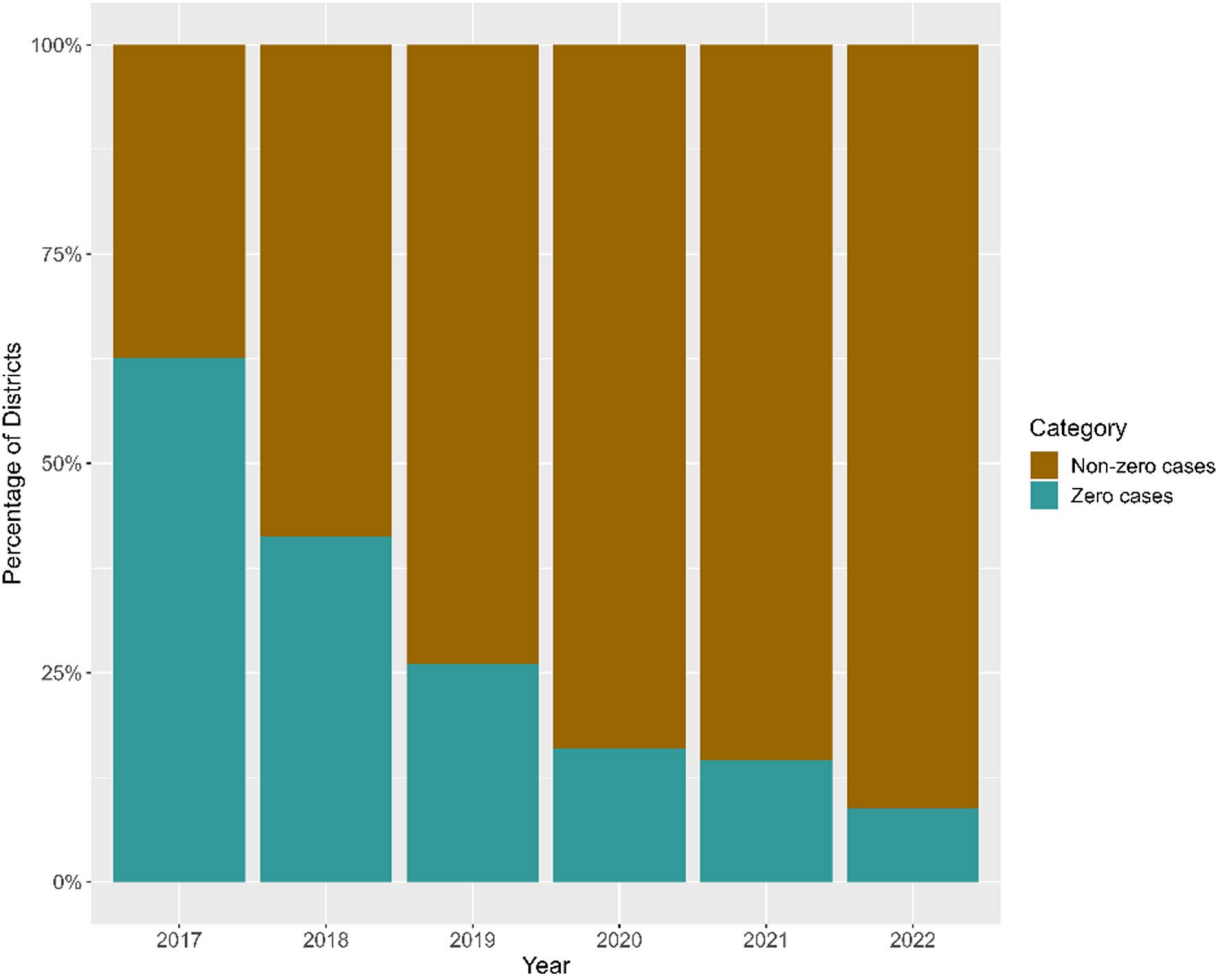
Stacked bar chart showing the annual proportion of districts reporting drug-resistant tuberculosis cases in Indonesia from 2017 to 2022, categorized by districts with zero and non-zero case notifications

Although our primary assumption for modeling district-level DR-TB cases was based on the standard Poisson distribution, we observed a potential issue of zero-inflation in the data. Specifically, the proportion of districts reporting zero DR-TB cases varied over time with a notable peak in 2017, followed by a decrease in zero-case districts as DR-TB cases gradually increased as shown in Fig. 1. It reflected the roll-out of GeneXpert during this period [37, 38]. To address this issue, we extended the Poisson model to a zero-inflated Poisson (ZIP) model to accommodate the excessive zeros by combining a point mass at zero with a base distribution for positive outcomes.

In this context, let *Y*_*it*_ represented the DR-TB case count in district *i* in year *t*. The generic structure of the ZIP model was expressed as ${Y}_{it}={y}_{it}\sim p\times {I}_{{y}_{it} = 0}$ + $(1-p)\times$ *f* (${y}_{it}|{\upmu }_{it}$) where *I* was the indicator function, $p$ was the probability of a true DR-TB case (indicating the likelihood of being at risk for DR-TB), and $f({y}_{it}|{\upmu }_{it}$) was the base probability distribution for positive outcomes, conditioned on the mean ${\upmu }_{it}$ and other model parameters. In this model, structural zeros arose in areas inherently unsuitable for DR-TB transmission (such as districts with no exposure or risk factors), while sampling zeros resulted from random chance within susceptible populations. The parameter $1-p$ reflected the “at-risk” probability, representing the likelihood of belonging to a subpopulation at risk for DR-TB [37–39].

We also considered Negative Binomial (NB) models to account for overdispersion, where the variance exceeded the mean—a limitation often observed with the Poisson distribution. Although Poisson models with random effects were commonly used in Bayesian disease mapping and could accommodate some extra-Poisson variation, the NB distribution offered a more flexible alternative when overdispersion was evident. To comprehensively explore model performance, we compared four specifications: Poisson, NB, ZIP, and zero-inflated Negative Binomial (ZINB), aimed to address both overdispersion and zero inflation. Model selection was guided by goodness-of-fit criteria to identify the most appropriate model for estimating associations with DR-TB incidence.

The ZIP and ZINB models allowed for the separate modelling of the zero probability and mean incidence components. The zero probability component was linked via a logit function: $logit (p)=\mathrm{log}\left(\frac{p}{1-p}\right)={\alpha }_{p0}$ and the mean incidence was linked to a linear predictor as $log \left(\upmu \right)= \theta = {\alpha }_{\upmu 0}+{E}_{it}+{\sum }_{j = 1}^{J}\upbeta{\mathrm{j}}\text{. X}{\mathrm{it}}{\mathrm{j}}+ {\theta }_{it}$. Here, ${\mathrm{X}}{\mathrm{itj}}$ represented spatiotemporal risk factors, including treatment coverage, treatment completion rate, treatment success rate, health centre density, insurance coverage, poverty rates, access to sanitation, and the municipal human development index. Different configurations of spatiotemporal random effects were explored to identify the best-fitting model. We focused on modelling the mean incidence component, which was directly linked to the observed outcomes to maintain and interpretability.

To construct spatial random effects, the Besag, York, and Mollié (BYM) model was employed. This model specified the area-level relative risks using two random effects: a spatially structured component ${u}_{i}$ and an unstructured component ${v}_{i}$ [40]. To account for spatial dependency in the district-level DR-TB notification data, the spatially structured random effect ${u}_{i}$ was modeled using the conditional autoregressive (CAR) model proposed by Besag et al. [41]. The CAR model specified $u_{i} |\boldsymbol{u}_{{ - i}} ~\sim Normal\left( {{{\bar{\mathrm{u}}}}_{{\Omega _{i} ~}} ,\frac{{\sigma _{u}^{2} }}{{n\delta _{i} }}} \right)$ where where ${\boldsymbol{u}}_{ - i}$ was the vector containing the correlated effect of all except the *i*th district. $\Omega_{i}$, $n_{{\delta_{i} }}$ and $\overline{u}_{{\delta_{i} }}$ were a set of the first-order spatial neighbours, cardinality and the average of the neighbourhood of the *i* th district respectively. Additionally, an unstructured random effect ${v}_{i}$ was assumed to follow a Gaussian prior distribution with zero mean and variance to account for heterogeneity beyond spatial autocorrelation.

To capture temporal dependencies, a temporal random effect ${T}_{t}$ was introduced with two configurations. The first configuration used a random walk of order 1 (RW1), assuming a dependency on the preceding year, defined as ${T}_{t}\sim Normal ({T}_{t-1}, {\sigma }_{u}^{2})$. The second configuration modeled temporal effects as independent and identically distributed (IID), specified as ${T}_{t}\sim Normal (0, {\sigma }_{u}^{2})$. In addition to spatial and temporal random effects, we added another term ${\delta }_{it}$ or spatial-temporal interaction type I to the model, representing unstructured space-time random effect interaction [42]. This interaction was assumed that both effects were flexible and unstructured. This interaction structure matrix was ${R}_{\delta }$ and was derived using the Kronecker product $\otimes$ with the identity matrix $I$. Consequently, the structure matrix was represented as ${R}_{\delta }= {R}_{v} \otimes {R}_{T}= I\otimes I = I$. Under these assumptions, we captured the space-time interaction as ${\delta }_{it}\sim Normal (0, {\sigma }_{\delta }^{2})$. Precision parameters, representing the inverse of variance, were modeled using a Log-Gamma distribution. For the CAR model, hyperparameters of 1 and 0.0005 were used, while hyperparameters of 1 and 0.00005 were applied to the unstructured and random walk random effects. Coefficients for fixed effects were modeled using a zero-mean Gaussian distribution with a precision of 0.001. We chose these weakly informative priors for the hyperparameters based on standard recommendations in the objective Bayesian framework. These priors were commonly used in spatial and spatiotemporal disease mapping to provide estimation while allowing the data to primarily inform the posterior distributions.

Recent comparative studies have shown that Bayesian spatiotemporal models with exceedance probability provide superior performance over traditional cluster detection methods [22, 23]. To identify districts with elevated DR-TB incidence, we employed exceedance probability estimation, assessing the likelihood that the observed disease risk in a district ${\upmu }_{it}/{E}_{it}$ exceeded a predefined threshold *q* [43]. The exceedance probability was computed as:

6 $$\mathit{Pr}({\upmu }_{it}/{E}_{it}>q)= 1- \alpha$$

where and ${\upalpha }= 0.05$ was level of significant threshold. To comprehensively explore various modelling assumptions regarding likelihood functions and space-time random effects, we developed a total of 80 model specifications to address the observed data structure. We computed various configurations of zero-inflation models, incorporating various combinations of space-time random effects in both the zero proportion and mean incidence components. These models aimed to identify the best-fitting model for understanding DR-TB incidence in Indonesia. Detailed descriptions of the model formulations and configurations were provided in supplementary document S3.

### Comparison and selection of model specification

To evaluate the performance of the model specifications, we used the Deviance Information Criterion (DIC) [44] and the Watanabe-Akaike Information Criterion (WAIC) for their ability to assess model goodness-of-fit while penalizing for model complexity in Bayesian modeling. Primarily, models with lower DIC and WAIC values were considered to have better performance, reflecting an optimal balance between model fit and complexity, and were prioritized for final interpretation [45–47]. However, in instances where DIC and WAIC values were comparable across models, we further assessed model complexity by examining the effective number of parameters for Deviance Information Criterion (*p*DIC) and parameters for Watanabe-Akaike Information Criterion (*p*WAIC) to support model selection.

To identify risk factors, significant variables for selected model were identified using the 95% credible intervals (CrI) of the posterior distributions, ensuring robust and reliable inferences about the associations between risk factors and DR-TB incidence. The Integrated Nested Laplace Approximation (INLA) method was used for model computation and evaluation, implemented via the R-INLA package (version 22.12.16; Rue et al. 2009). Analyses were conducted using RStudio (version 2023.06.0, R Core Team, Vienna, Austria).

## Results

### National trends and demographic patterns of notified DR-TB in Indonesia

**Fig. 2:**
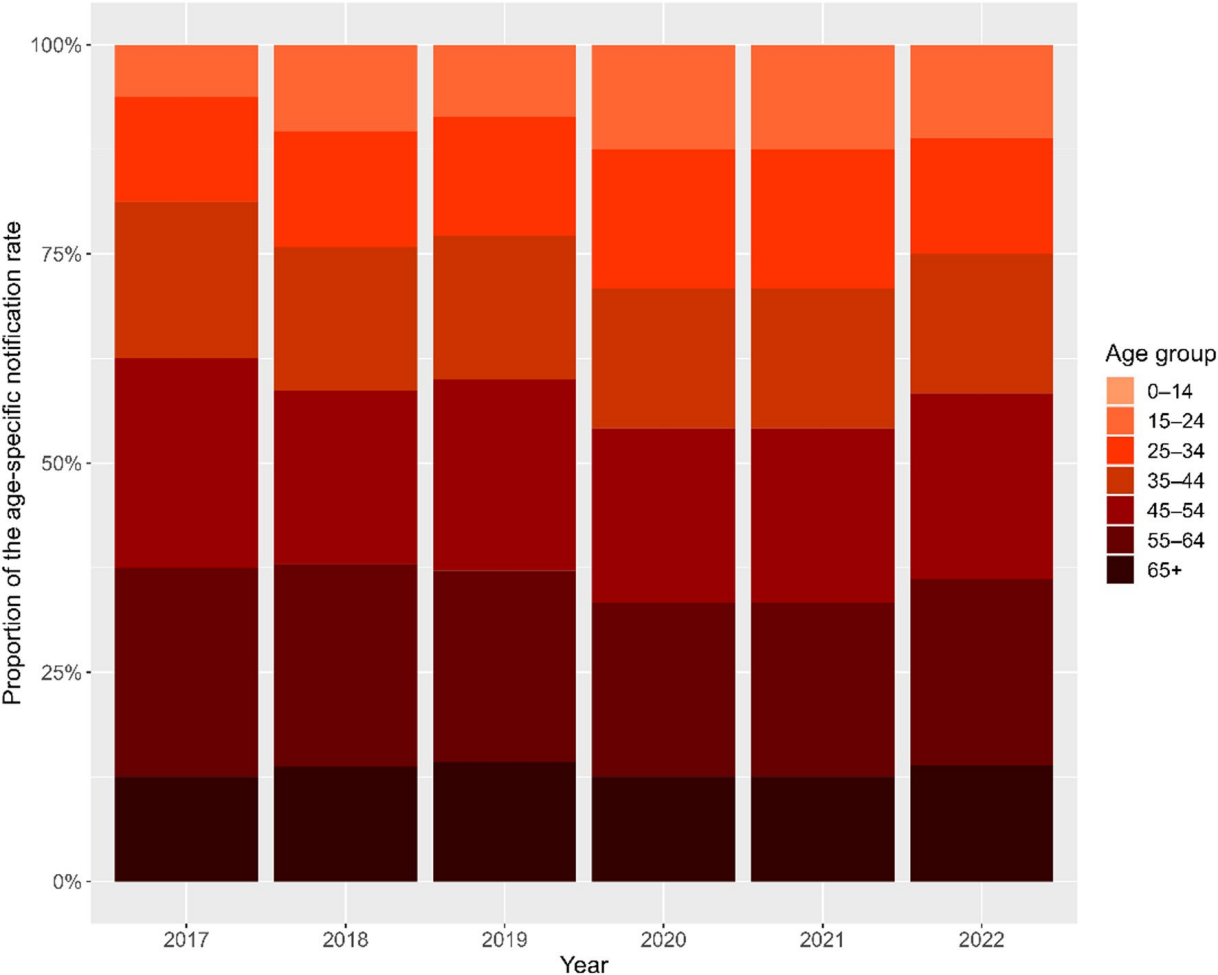
Bar chart illustrating the age-specific proportion of drug-resistant tuberculosis case notifications in Indonesia from 2017 to 2022, disaggregated by age group

During 2017–2022, a total of 54,291 DR-TB cases were notified in Indonesia, with approximately 75% classified as pulmonary TB. While notifications increased annually during the first three years, a noticeable decline occurred in 2020. Subsequently, notifications rebounded significantly, rising consistently through 2022. In 2017, only 37.4% of districts reported the cases, highlighting a significant proportion of districts with zero notifications. Despite a sharp decline in case numbers during 2020, DR-TB cases were still reported in 85% of districts and this proportion increased to 91.2% by 2022. Age-specific notification rates presented in Fig. 2, show that the working-age population (25–54 years) had higher DR-TB notification rates compared to children, teenagers, and elders. In 2017, the DR-TB notification rates for males and females were equal, both recorded at 2 per 100,000 population. However, from 2018 onward, the male notification rate consistently exceeded the female rate, reaching 5 per 100,000 male population in 2022 compared to 4 per 100,000 female population.

**Fig. 3:**
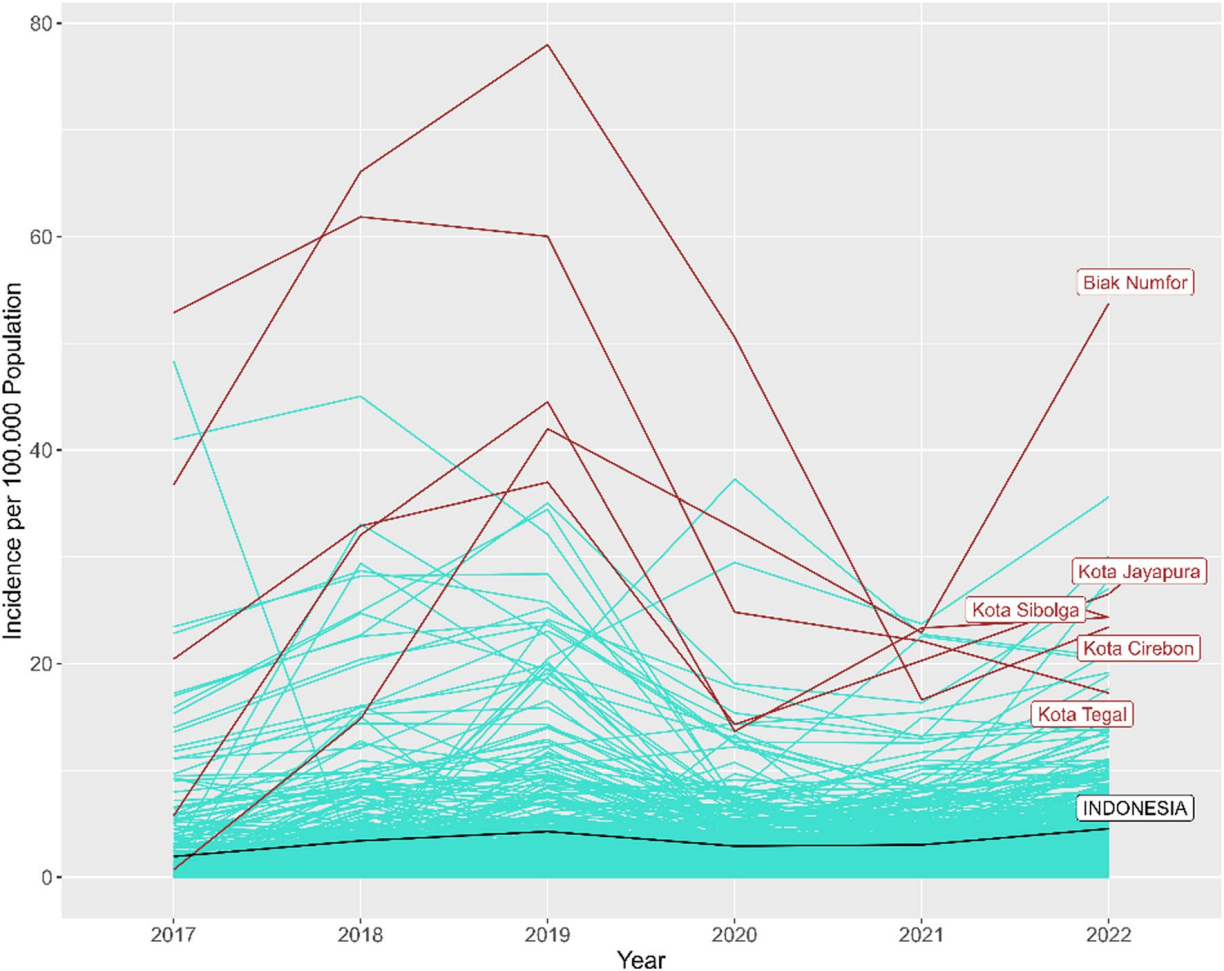
Line graph displaying drug-resistant tuberculosis reported cases per 100,000 population throughout all 514 districts during 2017–2022. The red lines represent the five highest-incidence districts, the blue lines represent incidence in other districts, and the black line indicates national incidence

We observed a significant increase in the national reported DR-TB rate in Indonesia, rising from 1.94 to 4.54 per 100,000 population over the study period, as illustrated in Fig. 3. Several districts had incidence rates significantly higher than the national average. Among the five districts with the highest average incidence, four were cities or “kota.” The top five districts were Cirebon City (45.23 per 100,000 population), Tegal City (39.81 per 100,000 population), Biak Numfor (27.81 per 100,000 population), Jayapura City (25.24 per 100,000 population), and Sibolga City (23.94 per 100,000 population).

### Geographical distribution and hotspots of DR-TB

**Fig. 4:**
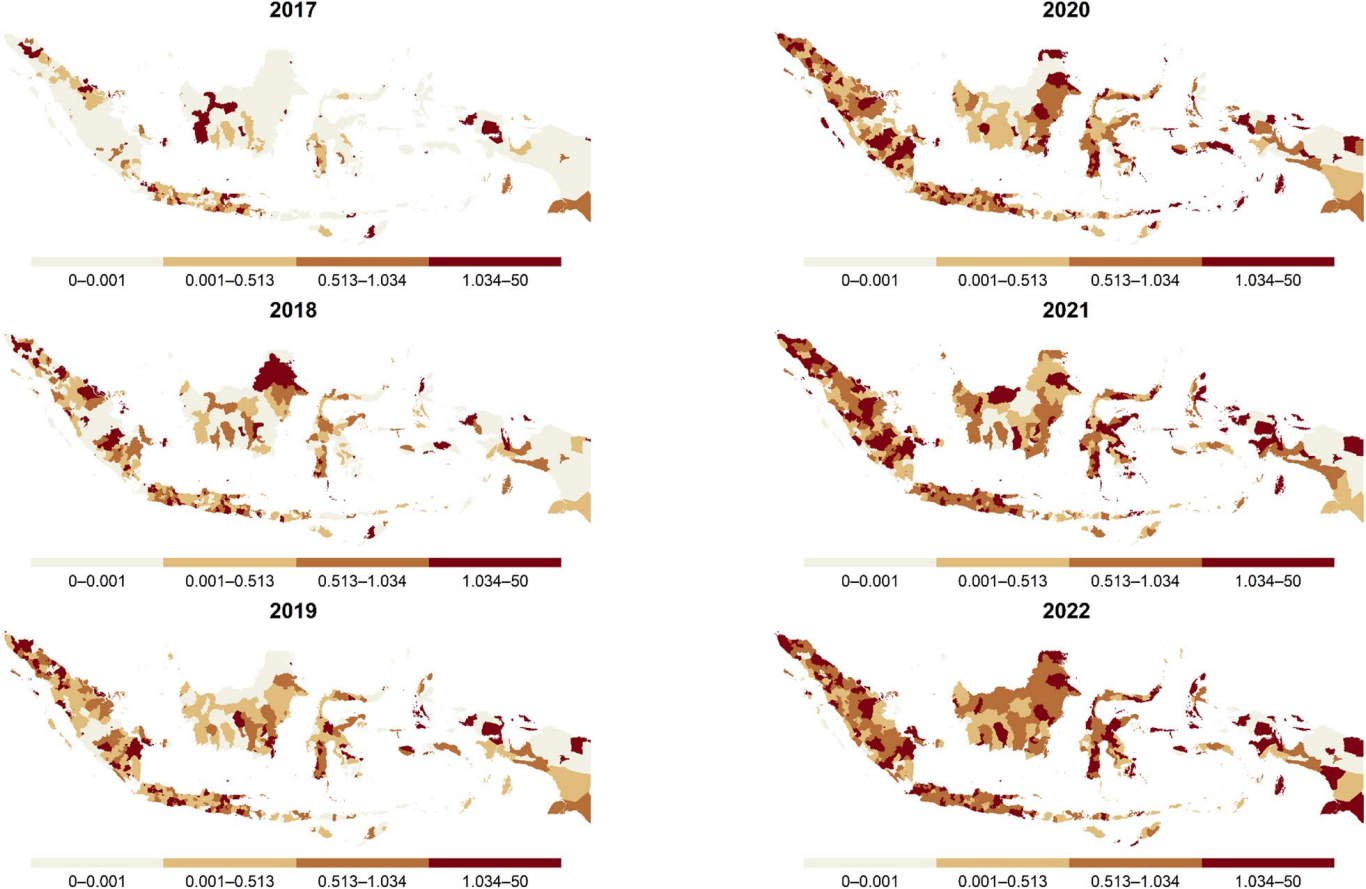
Spatiotemporal distribution of SIRs for DR-TB at the district level across Indonesia from 2017 to 2022. Districts are shaded with darker tones indicating higher incidence relative to the national average. *DR-TB* Drug-resistant tuberculosis; *SIR* Standardized incidence rate

Concerning spatiotemporal patterns of SIR, there was a significant spatial variability in 2017 which primarily concentrated on the island of Java (Fig. 4). However, nationwide heterogeneity was limited due to a high number of zero notifications. In 2017, the median SIR was 0.2, with most districts reporting SIRs below 0.5. By 2022, spatial heterogeneity was more pronounced across all provinces, with the median SIR rising to 0.79 and most districts reporting SIRs around 0.5.

**Fig. 5:**
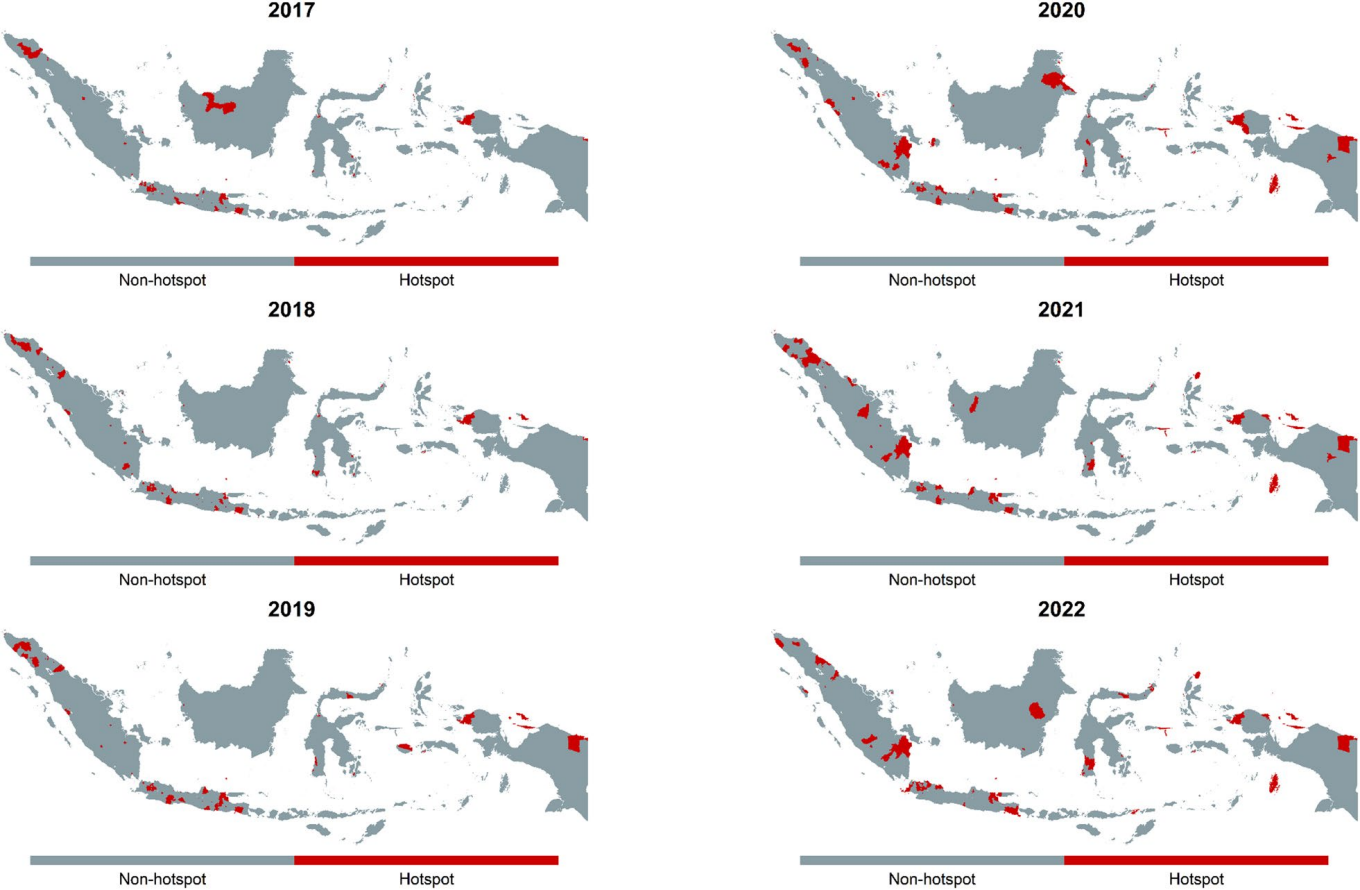
Spatiotemporal distribution of DR-TB hotspots across districts in Indonesia from 2017 to 2022. Districts classified as hotspots are highlighted in red, while non-hotspot areas are shown in grey. *DR-TB* Drug-resistant tuberculosis

We analyzed the spatiotemporal distribution of DR-TB hotspots to identify areas with risks exceeding expected rates depicted in Fig. 5. Over 2017–2022, hotspots were geographically spread across country, with a notable concentration in urbanized areas. Persistent hotspots formed clusters throughout the study, seen in Jakarta with its surrounding cities as the largest urban center, and in Greater Surabaya with its surroundings as the second largest. Additional hotspots were identified in Palembang city and its surroundings. Meanwhile, hotspots in some cities did not form clusters with their surroundings, such as Sorong, Jayapura, Cirebon, Tasikmalaya, Semarang, Jember, and Medan.

### Spatiotemporal model selection and associations with DR-TB incidence

We evaluated various modeling structures to explain the spatiotemporal patterns and risk factor associations of DR-TB incidence. Based on Table 1, the best-fitted models that produced lower evaluation metrics within their respective likelihood categories were models incorporating a complete random effect, consist of BYM model, temporal effects, and spatial-temporal interaction type 1. Models using standard Poisson likelihood exhibited the lowest DIC and WAIC among all likelihood, suggesting it may offer the most parsimonious fit for the data. Further details of the complete modelling results are provided in supplementary document S4.

**Table 1:** Summary of model evaluation under different likelihood assumptions, including fit statistics

Likelihood assumption	Specification	DIC	${\boldsymbol{p}}$ DIC	WAIC	${\boldsymbol{p}}$ WAIC
Poisson	$u+ {T}_{t}^{RW1}$	− 9.53 × 10^293^	− 9.53 × 10^293^	1,141,585.46	536,049
	$v+ {T}_{t}^{RW1}$	19,867.43	− 1355.31	37,286.87	8156.86
	$u+v+ {T}_{t}^{RW1}$	19,894.85	− 1338.76	37,030.36	8027.89
	$u+v+ {T}_{t}^{RW1}+ {\delta }_{it}$	14,515.08	2008.15	14,460.61	1412.55
	$u+ {T}_{t}$	− 4.46 × 10^294^	− 4.46 × 10^294^	560,030.13	244,309.1
	$v+ {T}_{t}$	19,861.77	−1358.20	37,327.68	8177.75
	$u+v+ {T}_{t}$	19,862.51	−1355.03	37,112.00	8069.08
	$u+v+ {T}_{t}+ {\delta }_{it}$	14,513.68	2006.17	14,461.57	1412.84
ZIP (vary across space and time)	$u+ {T}_{t}^{RW1}$	− 2.36 × 10^291^	− 2.36 × 10^291^	8,580,706.35	4,246,433
	$v+ {T}_{t}^{RW1}$	23,545.53	− 1350.08	40,752.58	8055.93
	$u+v+ {T}_{t}^{RW1}$	23,550.66	− 1343.35	40,710.54	8035.32
	$u+v+ {T}_{t}^{RW1}+ {\delta }_{it}$	18,181.12	2023.88	18,122.40	1419.21
	$u+ {T}_{t}$	− 3.99 × 10^291^	− 3.99 × 10^291^	1,382,235.12	653,758.4
	$v+ {T}_{t}$	23,508.04	− 1368.35	40,831.32	8096.19
	$u+v+ {T}_{t}$	23,530.48	− 1354.8	40,796.87	8077.92
	$u+v+ {T}_{t}+ {\delta }_{it}$	18,185.34	2011.53	18,134.75	1421.30
NB	$u+ {T}_{t}^{RW1}$	16,890.49	331.13	16,999.36	354.28
	$v+ {T}_{t}^{RW1}$	16,735.78	379.48	16,827.70	387.36
	$u+v+ {T}_{t}^{RW1}$	16,740.53	382.98	16,827.17	387.01
	$u+v+ {T}_{t}^{RW1}+ {\delta }_{it}$	16,659.68	659.73	16,705.48	581.04
	$u+ {T}_{t}$	16,890.02	331.18	16,998.89	354.37
	$v+ {T}_{t}$	16,735.69	380.56	16,827.34	388.02
	$u+v+ {T}_{t}$	16,739.65	383.56	16,827.63	388.20
	$u+v+ {T}_{t}+ {\delta }_{it}$	16,556.10	893.06	16,587.82	744.18
ZINB (vary across space and time)	$u+ {T}_{t}^{RW1}$	20,559.80	331.61	20,669.26	355.41
	$v+ {T}_{t}^{RW1}$	20,412.15	386.87	20,498.92	389.48
	$u+v+ {T}_{t}^{RW1}$	20,395.56	379.89	20,498.56	392.82
	$u+v+ {T}_{t}^{RW1}+ {\delta }_{it}$	20,204.02	752.95	20,306.01	691.17
	$u+ {T}_{t}$	20,561.12	333.83	20,669.10	355.97
	$v+ {T}_{t}$	20,407.84	375.44	20,503.48	386.94
	$u+v+ {T}_{t}$	20,404.05	383.72	20,500.64	391.05
	$u+v+ {T}_{t}+ {\delta }_{it}$	20,196.60	887.09	20,246.15	747.50

Abbreviations: *DIC* Deviance Information Criterion; *WAIC* Watanabe-Akaike Information Criterion; *ZIP* Zero-inflated Poisson; *NB* Negative Binomial; *ZINB* Zero-inflated Negative Binomial

We conducted a sensitivity analysis to evaluate the robustness of our association estimates among all best-fitted models in each respective likelihood. We noted that all optimal models, which exhibited similar magnitudes and directions of incidence rate ratio (IRR) for each factor, as shown in Table 2. Given that the Poisson model demonstrated the best goodness-of-fit, we interpret the IRR under this model assumption. Each model provides a 95% credible interval (CrI) for the IRR, indicating the range within which the true value is likely to fall with 95% probability. For further details of all estimated associations across all configured models, please refer to supplementary document S5.

**Table 2:** Incidence rate ratio estimates of covariates and evaluation metric values of the best models under model assumptions, shown with 95% credible intervals (CrI)

Risk factor variables	Incidence rate ratio (95% CrI)			
	Poisson model	Zero-inflated Poisson model	Negative binomial model	Zero-inflated negative binomial model
Treatment coverage	0.888 (0.763–1.031)	0.893 (0.769–1.038)	0.894 (0.769–1.039)	0.896 (0.774–1.038)
Treatment completion rate	2.435 (1.843–3.219)	2.355 (1.783–3.111)	2.458 (1.865–3.242)	2.485 (1.890–3.267)
Treatment success rate	0.508 (0.368–0.702)	0.513 (0.370–0.711)	0.520 (0.377–0.716)	0.513 (0.373–0.706)
Health center per 100,000 population	0.986 (0.970–1.002)	0.985 (0.969–1.001)	0.992 (0.976–1.008)	0.981 (0.965–0.997)
Proportion of insured population	1.001 (1.000–1.002)	1.001 (1.000–1.002)	1.001 (1.000–1.002)	1.001 (1.000–1.002)
Proportion of population below poverty	1.028 (1.013–1.044)	1.027 (1.013–1.041)	1.027 (1.013–1.041)	1.030 (1.017–1.044)
Proportion of households with access to sanitation	1.006 (1.002–1.010)	1.006 (1.002–1.010)	1.005 (1.001–1.009)	1.007 (1.003–1.011)
Municipal human development index	1.068 (1.049–1.094)	1.066 (1.047–1.086)	1.075 (1.056–1.094)	1.073 (1.054–1.092)
Evaluation measures				
DIC (*p*DIC)	14,513.68 (2006.17)	18,181.12 (2023.88)	16,556.10 (893.06)	20,196.60 (887.09)
WAIC (*p*WAIC)	14,461.57 (1412.84)	18,122.40 (1419.21)	16,587.82 (744.18)	20,246.15 (747.50)

Abbreviations: *DIC* Deviance Information Criterion; *WAIC* Watanabe-Akaike Information Criterion

From the Bayesian spatial regression modelling, we found that district IRR of DR-TB were associated with treatment program, health system, and socioeconomic factors in some ways as seen in Table 2. A notable finding from treatment program showed that treatment success rate had IRR below 1, with 95% CrIs ranging from 0.368 to 0.702. This association suggested that a one-unit increase in the treatment success rates was associated with 49.2% reduction in DR-TB incidence. Treatment completion rate showed IRR consistently above 2, with 95% CrIs ranging from 1.843–3.219, indicating a strong association between higher treatment completion rates and increased DR-TB incidence.

In the health system representing UHC, proportion of the insured population had IRR around 1, with 95% CrIs ranging from 1.000 to 1.002, suggesting an absence to little effect on increased DR-TB incidence. Regarding socioeconomic variables, proportion of the population below the poverty line had IRR above 1, with 95% CrIs ranging from 1.013 to 1.044, indicating that a one-unit increase in the proportion of the population below the poverty line was associated with a 2.8% increase in DR-TB incidence. In addition, there was a modest association between improved sanitation access and increased DR-TB incidence also shown as the IRR were slightly above 1, with 95% CrIs ranging from 1.002 to 1.010. This ratio indicated that a one-unit increase in the proportion of households with access to sanitation was associated with a 0.6% rise in DR-TB incidence. Lastly, the municipal human development index had IRR above 1, with 95% CrIs ranging from 1.049 to 1.094, indicating that a one-unit increase in MHDI was associated with a 6.8% growth in DR-TB incidence.

Nevertheless, some covariates had no association with DR-TB incidence. Treatment coverage exhibited an IRR slightly below 1, with 95% CrIs ranging from 0.763 to 1.031. Despite this suggesting a modest reduction in DR-TB incidence with increased treatment coverage, the evidence is not strong due to the CrIs encompassing 1. In the health system variable, health center density had IRR close to 1, with 95% CrIs ranging from 0.970 to 1.002, indicating a minimal effect on DR-TB incidence.

## Discussion

This nationwide study analyzed spatiotemporal patterns and risk factors associated with DR-TB across 514 districts in Indonesia from 2017–2022. DR-TB incidence rose between 2017 and 2019, declined during the first year of the COVID-19 pandemic, and increased again thereafter. Most cases occurred among individuals aged 25–54 years, corresponding to the working-age population. Hotspots were concentrated in major urban centers such as Jakarta and Surabaya. Higher treatment success rates were associated with reduced DR-TB incidence, whereas higher treatment completion rates, poverty, improved sanitation, and higher MHDI were linked to increased incidence.

The temporary decline in DR-TB incidence during 2020 might have occurred with disruptions in TB control activities caused by the COVID-19 pandemic. Disruptions to TB diagnostic services likely reduced case detection and reporting, which in turn contributed to lower treatment initiation due to reduced patient access to health facilities. Surendra et al. reported a 26% drop in case notifications and an 11% decline in treatment coverage, with gradual recovery from 2021 onward [48]. Meanwhile, the number of districts reporting DR-TB increased markedly—from 37% in 2017 to 91% in 2022—largely due to the expansion of GeneXpert MTB/RIF diagnostic coverage from 495–1809 machines nationwide [28]. This expansion likely improved detection and contributed to the spatial heterogeneity observed in this study.

Model comparisons using Poisson, NB, and zero-inflated likelihoods showed that the standard Poisson model with space–time random effects provided the best fit, consistent with findings from previous Bayesian surveillance studies [46, 47]. Sensitivity analyses confirmed stable IRR estimates across model specifications, suggesting robustness of the identified associations. The inclusion of spatiotemporal interaction terms improved model performance by capturing unobserved heterogeneity and local clustering effects.

Urban clustering of DR-TB cases reflects both increased diagnostic capacity and the epidemiological influence of urbanization. High population mobility and migration in densely populated cities facilitate transmission [49]. Similar findings in Ukraine showed that urban residents had 37% higher odds of rifampicin-resistant TB than rural populations [50]. Urban areas may also face challenges in treatment adherence due to demanding work schedules [51]. Workplace-based interventions—such as flexible treatment hours, on-site directly observed treatment (DOT) programs, and stigma reduction initiatives—are therefore essential to improve adherence [52].

Our results also underscore weaknesses in using self-reported treatment completion as a performance indicator. While completion rates improved nationally from 43–63% during the study period, this metric may overestimate adherence due to social desirability and recall biases [53, 54]. In contrast, treatment success rate—defined by bacteriological confirmation of cure—was inversely associated with DR-TB incidence, emphasizing its importance as a more reliable indicator for monitoring treatment outcomes. These findings reinforce the need to strengthen adherence verification through objective measures such as biomarker testing and enhanced patient monitoring.

Poverty remains a consistent driver of DR-TB incidence. Economic hardship contributes to poor nutrition, weak immunity, and delayed healthcare seeking [55, 56], while DR-TB itself reinforces poverty by reducing individual and household productivity—through loss of employment, diminished work capacity, prolonged treatment duration, and high out-of-pocket healthcare costs [56, 57]. Conversely, the positive associations with sanitation access and MHDI may reflect urbanicity rather than causal effects, as better infrastructure coincides with denser populations and greater diagnostic access [28, 58, 59]. Targeted support for vulnerable groups in urban settings—particularly low-income and migrant workers—is critical to mitigate these disparities.

Despite its strengths, this study has limitations. Reliance on notification data may underestimate true DR-TB burden, as undiagnosed and underreported cases likely contribute to an excess of zero counts. These zero inflations may reflect false absences rather than true zero incidence and can influence the choice and performance of statistical models used in the analysis. Nonetheless, notified data remain a standardized source within the health system, despite findings that 25% of laboratory-confirmed DR-TB cases were missing from treatment registers in Sumatra [60] and only 9.8% of estimated cases were diagnosed in a tertiary hospital [61]. In addition, incomplete SITB entries limited distinction between primary and acquired resistance. Potential collinearity between sanitation access and MHDI may also reflect urbanicity effects. As an ecological analysis, the results represent area-level associations rather than individual-level causality and may have been unable to capture all relevant confounders. Despite these constraints, the findings provide actionable evidence to guide targeted interventions and strengthen DR-TB surveillance in Indonesia.

## Conclusions

This study provides the first nationwide district-level assessment of DR-TB epidemiology in Indonesia, revealing spatial and temporal heterogeneity shaped by diagnostic expansion, socioeconomic disparities, and treatment performance. Strengthening surveillance, healthcare access, and implementing surveillance and active case finding in incidence hotspots could enhance early detection and enrollment into treatment. To reduce mortality, TB control programs should prioritize improving treatment success rates rather than nominal completion rates, ensuring true cure outcomes through better adherence monitoring. Finally, integrating socioeconomic and workplace-based interventions can help address the persistent inequities driving DR-TB in urban settings, supporting Indonesia’s goal to eliminate TB by 2030.

## Supplementary Information

Additional file 1.

## Data Availability

The data supporting the findings of this study were obtained from Indonesia's National Tuberculosis Program through the *Sistem Informasi Tuberkulosis* (SITB). Access to these data is restricted and was granted solely for the purposes of this study; therefore, the data are not publicly available.

## Abbreviations

BYM: Besag, York, and Mollié
CAR: Conditional Autoregressive
CrI: Credible Interval
DIC: Deviance Information Criterion
DR-TB: Drug-resistant tuberculosis
IID: Independent and Identically Distributed
INLA: Integrated Nested Laplace Approximation
IRR: Incidence Rate Ratio
LPA: Line Probe Assay
MDR-TB: Multidrug-resistant tuberculosis
MHDI: Municipal Human Development Index
RR-TB: Rifampicin-resistant tuberculosis
RW1: Random Walk of order 1
SITB: Sistem Informasi Tuberculosis
SIR: Standardized Incidence Rate
STROBE: Strengthening the reporting of observational studies in epidemiology
UHC: Universal Health Coverage
WAIC: Watanabe-Akaike Information Criterion
ZINB: Zero-inflated Negative Binomial
ZIP: Zero-inflated Poisson
