# Supplementary material for: Spatiotemporal epidemiology, geographic hotspots, and risk factor associations of drug-resistant tuberculosis incidence in Indonesia: a Bayesian hierarchical modelling approach
Source: Infect Dis Poverty. 2026 Feb 13;15:23. doi: 10.1186/s40249-026-01418-9 (PMC12903336; doi:10.1186/s40249-026-01418-9)
Supplement: Supplementary file 1 — Additional file 1. [file 40249_2026_1418_MOESM1_ESM.docx]

**Spatiotemporal epidemiology, geographic hotspots, and risk factor associations of drug-resistant tuberculosis incidence in Indonesia: a Bayesian hierarchical modelling approach**

**Supplementary document S1: Data sources and operational definitions of indicators used in the study**

| **No** | **Covariates** | **Definition** | **Source** |
| --- | --- | --- | --- |
| 1 | Number of Drug-resistant Tuberculosis | Number of TB patients that are resistant to at least one first-line anti-TB drug | National Tuberculosis Program, Ministry of Health |
| 2 | TB treatment coverage | Proportion of TB patients who start the treatment among all estimated TB cases | National Tuberculosis Program, Ministry of Health |
| 3 | TB treatment complete rate | Proportion of TB patients who completed their prescribed treatment regimen | National Tuberculosis Program, Ministry of Health |
| 4 | TB treatment success rate | Proportion of notified TB patients who complete their prescribed treatment regimen and have no bacteriological evidence of ongoing infection at follow-up | National Tuberculosis Program, Ministry of Health |
| 5 | Health center per 100,000 population | Number of public health centers (registered in NTP which serve DR-TB treatment) per 100,000 population | National Statistical Agency |
| 6 | Universal Health Coverage | Proportion of population that is covered by national health insurance system (contributory and non-contributory schemes) | National Agency for Health Security |
| 7 | Poverty headcount index | Percentage of the population with per capita expenditure below the poverty line | National Statistical Agency |
| 8 | Access to sanitation | Percentage of households which have good access to sanitation and are using improved sanitation facilities | National Statistical Agency |
| 9 | Municipal Human Development Index | A localized version of the Human Development Index (HDI) that quantifies a region’s average achievements in basic human development | National Statistical Agency |

**Table S1.1 Data sources and operational definitions of indicators used in the study.**

**Supplementary document S2: STROBE checklist**

| **Section** | **Item no** | **Recommendation** | **Checklist note** |
| --- | --- | --- | --- |
| **TITLE and ABSTRACT** | 1 | Indicate the study’s design with a commonly used term in the title or the abstract | We already provided the study design in the title: A Bayesian Hierarchical Modelling |
|  |  | Provide in the abstract an informative and balanced summary of what was done and what was found | Summary of the method (what was done) and results (what was found) has been provided in the abstract |
| **INTRODUCTION** |  |  |  |
| Background / rationale Objectives | 2 | Explain the scientific background and rationale for the investigation being reported | The background consists of: 1) global and national burden of disease; 2) methodological gaps; 3) objectives |
|  | 3 | State specific objectives, including any prespecified hypotheses |  |
| **METHODS** |  |  |  |
| Study design | 4 | Present key elements of study design early in the paper | Study design, data collection, and analysis were presented in the abstract (early), and briefly detailed in methods section |
| Setting | 5 | Describe the setting, locations, and relevant dates, including periods of recruitment, exposure, follow-up, and data collection | The setting, locations, and relevant dates have been clearly stated in method section |
| Participants | 6 | 1. Cohort study—Give the eligibility criteria, and the sources and methods of selection of participants. Describe methods of follow-up   Case-control study—Give the eligibility criteria, and the sources and methods of case ascertainment and control selection. Give the rationale for the choice of cases and controls  Cross-sectional study—Give the eligibility criteria, and the sources and methods of selection of participants   1. Cohort study—For matched studies, give matching criteria and number of exposed and unexposed   Case-control study—For matched studies, give matching criteria and the number of controls per case | This was a retrospective study with secondary data analysis procedures.  We did not include process to involve selected participant. |
| Variables | 7 | Clearly define all outcomes, exposures, predictors, potential confounders, and effect modifiers. Give diagnostic criteria, if applicable | In the methods we have provided outcomes and predictors; while potential confounders were mentioned in the limitation section |
| Data sources / measurement | 8* | For each variable of interest, give sources of data and details of methods of assessment (measurement).  Describe comparability of assessment methods if there is more than one group | All variables and its definition, including the sources were clearly mentioned in method section. |
| Bias | 9 | Describe any efforts to address potential sources of bias | Potential sources of bias were addressed in both the discussion and limitations sections of the manuscript. |
| Study size | 10 | Explain how the study size was arrived at | The nationwide scope of this study was consistently emphasized throughout the manuscript, including in the abstract, background, methods, and discussion sections. |
| Quantitative variables | 11 | Explain how quantitative variables were handled in the analyses. If applicable, describe which groupings were chosen, and why | The outcome variable, reported as notification counts, was treated as a quantitative variable and incorporated into the analysis using mathematical formulations to compute SIRs and identify spatial and temporal hotspots |
| Statistical methods | 12 | 1. Describe all statistical methods, including those used to control for confounding 2. Describe any methods used to examine subgroups and interactions 3. Explain how missing data were addressed 4. Cohort study—If applicable, explain how loss to follow-up was addressed   Case-control study—If applicable, explain how matching of cases and controls was addressed  Cross-sectional study—If applicable, describe analytical methods taking account of sampling strategy   1. Describe any sensitivity analyses | 1. Several statistical methods were clearly described in the Methods section to ensure transparency and reproducibility. 2. We examined potential interactions between DR-TB incidence and selected risk factors through ecological regression analysis to explore their associations. 3. The institutional data used in this study were complete and did not contain any missing values. 4. As this was a retrospective analysis using nationwide data, no sampling strategy was applied. 5. Sensitivity analysis was conducted using model selection criteria to compare the performance of alternative models and confirm the robustness of the findings. |
| **RESULTS** |  |  |  |
| Participants | 13* | 1. Report the numbers of individuals at each stage of the study—e.g., numbers potentially eligible, examined for eligibility, con-firmed eligible, included in the study, completing follow-up, and analysed 2. Give reasons for non-participation at each stage 3. Consider use of a flow diagram | 1. We reported 54,291 DR-TB cases in the Results, but these are not study participants since we used retrospective aggregated data. 2. This study did not involve individual participants, as it used routine surveillance data. 3. A flow diagram was not used because the study did not include recruitment or follow-up. Instead, we used mathematical notation to describe the analysis. |
| Descriptive data | 14* | 1. Give characteristics of study participants (e.g., demographic, clinical, social) and information on exposures and potential con-founders 2. Indicate the number of participants with missing data for each variable of interest 3. Cohort study—Summarise follow-up time (e.g., average and total amount) | 1. The methods section describes the study characteristics, including the unit of analysis (district-year), predictor variables, and outcome (DR-TB notification counts) 2. The outcome variable was complete. Minor missing values in covariates were addressed using linear interpolation. 3. Not applicable, as this was a retrospective ecological study without individual follow-up |
| Outcome data | 15* | Cohort study—Report numbers of outcome events or summary measures over time  Case-control study—Report numbers in each exposure category, or summary measures of exposure  Cross-sectional study—Report numbers of outcome events or summary measures | In our outcome data, we reported annual DR-TB notification counts and derived district-level notification rates over six years (2017–2022) to summarize outcome distribution across time and space. |
| Main results | 16 | 1. Give unadjusted estimates and, if applicable, confounder-adjusted estimates and their precision (e.g., 95% confidence interval). Make clear which confounders were adjusted for and why they were included. 2. Report category boundaries when continuous variables were categorized 3. If relevant, consider translating estimates of relative risk into absolute risk for a meaningful time period | 1. We reported relative risk estimates (posterior means with 95% credible intervals) obtained from Bayesian ecological regression, adjusted for independent variables selected based on programmatic relevance and prior evidence. 2. All quantitative variables were treated as continuous and were not categorized. 3. Relative risks were not translated into absolute risks due to the ecological study design and the use of aggregated district-level data. |
| Other analyses | 17 | Report other analyses done—e.g., analyses of subgroups and interactions, and sensitivity analyses | We conducted sensitivity analyses by comparing alternative model specifications, which yielded consistent estimates. Potential interactions between key covariates were explored using Poisson regression. Descriptive subgroup analyses were also performed to examine age-specific and gender-specific notification rates. |
| **DISCUSSION** |  |  |  |
| Key results | 18 | Summarise key results with reference to study objectives | The summary of key results was provided in the first paragraph of the discussion. |
| Limitations | 19 | Discuss limitations of the study, taking into account sources of potential bias or imprecision. Discuss both direction and magnitude of any potential bias | In the Discussion section, we identified several limitations, including potential bias and confounding, data availability constraints, and possible collinearity. The direction and magnitude of their potential impact have also been addressed following each limitation. |
| Interpretation | 20 | Give a cautious overall interpretation of results considering objectives, limitations, multiplicity of analyses, results from similar studies, and other relevant evidence | The interpretation addressed the study objectives, focusing on spatial patterns, risk factor associations (without causal claims), relevant international findings, methodological strengths, and key limitations. |
| Generalisability | 21 | Discuss the generalisability (external validity) of the study results | We have addressed the generalisability of our findings in the discussion section, while emphasizing the need for caution due to context-specific differences. |
| **OTHER INFORMATION** |  |  |  |
| Funding | 22 | Give the source of funding and the role of the funders for the present study and, if applicable, for the original study on which thepresent article is based | We have included the funding agency in the acknowledgements section at the end of the manuscript. |

**Table S2.1** STROBE guideline checklist.

**Supplementary document S3: Zero-inflated model specifications in DR-TB notification data**

In our analysis of drug-resistant tuberculosis (DR-TB) notification data, we developed 80 distinct model specifications to elucidate the association between risk factors and DR-TB incidence. These models encompassed both Poisson and Negative Binomial likelihood functions, with 16 models based on standard Poisson and Negative Binomial distributions (eight each), and the remaining 64 models incorporating zero-inflation (ZI) components.

For the zero-inflated models, we explored various configurations of spatial and temporal random effects in both the zero-probability and mean incidence components. Specifically, we considered four scenarios for the zero-probability parameter ($p_{it}$):

1. Constant across space and time: $p_{it}=p$ for all locations and time periods, implying no random effects in the zero-probability component.
2. Varying across space, constant over time: $p_{it}=p_{i}$ for all time periods, allowing for spatial heterogeneity.
3. Constant across space, varying over time: $p_{it}=p_{t}$​ for all locations, capturing temporal variability.
4. Varying across space and time: $p_{it}=p_{it}$for all locations and time periods, accommodating both spatial and temporal variations.

To assess the relationship between district-level DR-TB incidence and covariates, we employed Bayesian spatiotemporal modeling under different distributional assumptions, including Poisson, zero-inflated Poisson (ZIP) with the aforementioned configurations, Negative Binomial, and zero-inflated Negative Binomial (ZINB) models. The ZIP and ZINB models are particularly suited for count data with excess zeros, as they combine a point mass at zero with a count distribution for positive outcomes.

The general formulation for the zero-inflated models is as follows:

- Zero-Inflated Poisson (ZIP):

$$y_{it}{\sim ZIP (\mu}_{it}, E_{it})$$

$$Pr\left( y_{it}=0 \mid\ldots\right)=p_{it}+\left( 1-p_{it} \right)\times exp\left( -\mu_{it} \right)$$

$$Pr \left( y_{it}=y_{it} \mid\ldots\right)=\left( 1-p_{it} \right)\times\frac{\mu_{it}^{y}\exp\left( -\mu_{it} \right)}{y_{it}!}, for y_{it}>0$$

- Zero-Inflated Negative Binomial (ZINB):

*Pr (*$y_{it}$*) =* $\left\{ \begin{aligned} p_{it}+\left( 1-p_{it} \right)\left( \frac{k}{k+\mu_{it}} \right)^{k} \mathrm{for} y_{it}=0 \\ \left( 1-p_{it} \right)\frac{\Gamma\left( y_{it}+k \right)}{\Gamma\left( k \right) \Gamma\left( y_{it}+1 \right)}\left( \frac{k}{k+\mu_{it}} \right)^{k}\left( \frac{k}{k+\mu_{it}} \right)^{y_{it}} \mathrm{for} y_{it}\geq0 \end{aligned} \right.$

Here, $\mu_{it}$ represents the mean incidence rate, $E_{it}$is the expected count, and *k* is the dispersion parameter for the Negative Binomial distribution. The parameter $p_{it}$denotes the zero-inflation probability, which varies according to the specified scenario.

By systematically varying these model components, we aimed to identify the most appropriate modeling framework for capturing the complexities of DR-TB incidence and its association with various risk factors. This comprehensive approach allowed us to account for overdispersion and excess zeros in the data, leading to more robust and reliable inferences.

In the first scenario set, the probability of zeros was assumed to be constant across space and time, so it was assumed that $p_{it}=p$for all locations and time periods, and there is no influence of random effect in zero-probability. Then the process of model was formulated as follow:

Model 9 (Poisson) and Model 49 (Negative binomial): the mean incidence with structured random effect and random walk of order 1 temporal effect

$logit \left( p_{it} \right)=logit(p)=\log\left( \frac{p}{1-p} \right)=\alpha_{p0}$ across locations and times.

$$log \left( \mu\right)= \theta= \alpha_{\mu0}+E_{it}+\sum_{j=1}^{J} \text{β}\text{j}\text{. X}\text{it}\text{j}+ u_{i}+T_{t}^{RW1}$$

Model 10 (Poisson) and Model 50 (Negative binomial): the mean incidence with unstructured random effect and random walk of order 1 temporal effect

$logit \left( p_{it} \right)=logit(p)=\log\left( \frac{p}{1-p} \right)=\alpha_{p0}$ across locations and times.

$$log \left( \mu\right)= \theta_{i}= \alpha_{\mu0}+E_{it}+\sum_{j=1}^{J} \text{β}\text{j}\text{. X}\text{it}\text{j}+ v_{i}+T_{t}^{RW1}$$

Model 11 (Poisson) and Model 51 (Negative binomial): the mean incidence with BYM random effect and random walk of order 1 temporal effect

$logit \left( p_{it} \right)=logit(p)=\log\left( \frac{p}{1-p} \right)=\alpha_{p0}$ across locations and times.

$$log \left( \mu\right)= \theta_{i}= \alpha_{\mu0}+E_{it}+\sum_{j=1}^{J} \text{β}\text{j}\text{. X}\text{it}\text{j}+ u_{i}+v_{i}+T_{t}^{RW1}$$

Model 12 (Poisson) and Model 52 (Negative binomial): the mean incidence with BYM random effect with spatial-temporal interaction term Type 1, and random walk of order 1 temporal effect

$logit \left( p_{it} \right)=logit(p)=\log\left( \frac{p}{1-p} \right)=\alpha_{p0}$ across locations and times.

$$log \left( \mu\right)= \theta_{i}= \alpha_{\mu0}+E_{it}+\sum_{j=1}^{J} \text{β}\text{j}\text{. X}\text{it}\text{j}+ u_{i}+v_{i}+T_{t}^{RW1}+\delta_{it}$$

Model 13 (Poisson) and Model 53 (Negative binomial): the mean incidence with structured random effect and unstructured temporal effect

$logit \left( p_{it} \right)=logit(p)=\log\left( \frac{p}{1-p} \right)=\alpha_{p0}$ across locations and times.

$$log \left( \mu\right)= \theta_{i}= \alpha_{\mu0}+E_{it}+\sum_{j=1}^{J} \text{β}\text{j}\text{. X}\text{it}\text{j}+ u_{i}+T_{t}$$

Model 14 (Poisson) and Model 54 (Negative binomial): the mean incidence with unstructured random effect and unstructured temporal effect

$$logit (p)=\log\left( \frac{p}{1-p} \right)=\alpha_{p0}$$

$$log \left( \mu\right)= \theta_{i}= \alpha_{\mu0}+E_{it}+\sum_{j=1}^{J} \text{β}\text{j}\text{. X}\text{it}\text{j}+ v_{i}+T_{t}$$

Model 15 (Poisson) and Model 55 (Negative binomial): the mean incidence with BYM random effect and unstructured temporal effect

$$logit (p)=\log\left( \frac{p}{1-p} \right)=\alpha_{p0}$$

$$log \left( \mu\right)= \theta_{i}= \alpha_{\mu0}+E_{it}+\sum_{j=1}^{J} \text{β}\text{j}\text{. X}\text{it}\text{j}+ u_{i}+v_{i}+T_{t}$$

Model 16 (Poisson) and Model 56 (Negative binomial): the mean incidence with BYM random effect with spatial-temporal interaction term Type 1, and unstructured temporal effect

$$logit (p)=\log\left( \frac{p}{1-p} \right)=\alpha_{p0}$$

$$log \left( \mu\right)= \theta_{i}= \alpha_{\mu0}+E_{it}+\sum_{j=1}^{J} \text{β}\text{j}\text{. X}\text{it}\text{j}+ u_{i}+v_{i}+T_{t}+\delta_{it}$$

In the second condition, the probability of zeros was assumed to be vary across space but constant over time. Thus, it was assumed that $p_{it}=p_{i}$for all time periods. Given the IID spatially unstructured random effect $v_{i}$ for zero-probability, the model variation can be constructed as follow:

Model 17 (Poisson) and Model 57 (Negative binomial): the mean incidence with structured random effect and random walk of order 1 temporal effect

$$logit {(p}_{i})=\log\left( \frac{p_{i}}{1-p_{i}} \right)=\alpha_{p0}+v_{i}$$

$$log \left( \mu_{i} \right)= \theta_{i}= \alpha_{\mu0}+E_{it}+\sum_{j=1}^{J} \text{β}\text{j}\text{. X}\text{it}\text{j}+ u_{i}+T_{t}^{RW1}$$

Model 18 (Poisson) and Model 58 (Negative binomial): the mean incidence with unstructured random effect and random walk of order 1 temporal effect

$$logit {(p}_{i})=\log\left( \frac{p_{i}}{1-p_{i}} \right)=\alpha_{p0}+v_{i}$$

$$log \left( \mu_{i} \right)= \theta_{i}= \alpha_{\mu0}+E_{it}+\sum_{j=1}^{J} \text{β}\text{j}\text{. X}\text{it}\text{j}+ v_{i}+T_{t}^{RW1}$$

Model 19 (Poisson) and Model 59 (Negative binomial): the mean incidence with BYM random effect and random walk of order 1 temporal effect

$$logit {(p}_{i})=\log\left( \frac{p_{i}}{1-p_{i}} \right)=\alpha_{p0}+v_{i}$$

$$log \left( \mu_{i} \right)= \theta_{i}= \alpha_{\mu0}+E_{it}+\sum_{j=1}^{J} \text{β}\text{j}\text{. X}\text{it}\text{j}+ u_{i}+v_{i}+T_{t}^{RW1}$$

Model 20 (Poisson) and Model 60 (Negative binomial): the mean incidence with BYM random effect with spatial-temporal interaction term Type 1, and random walk of order 1 temporal effect

$$logit {(p}_{i})=\log\left( \frac{p_{i}}{1-p_{i}} \right)=\alpha_{p0}+v_{i}$$

$$log \left( \mu_{i} \right)= \theta_{i}= \alpha_{\mu0}+E_{it}+\sum_{j=1}^{J} \text{β}\text{j}\text{. X}\text{it}\text{j}+ u_{i}+v_{i}+T_{t}^{RW1}+\delta_{it}$$

Model 21 (Poisson) and Model 61 (Negative binomial): the mean incidence with structured random effect and unstructured temporal effect

$$logit {(p}_{i})=\log\left( \frac{p_{i}}{1-p_{i}} \right)=\alpha_{p0}+v_{i}$$

$$log \left( \mu_{i} \right)= \theta_{i}= \alpha_{\mu0}+E_{it}+\sum_{j=1}^{J} \text{β}\text{j}\text{. X}\text{it}\text{j}+ u_{i}+T_{t}$$

Model 22 (Poisson) and Model 62 (Negative binomial): the mean incidence with unstructured random effect and unstructured temporal effect

$$logit {(p}_{i})=\log\left( \frac{p_{i}}{1-p_{i}} \right)=\alpha_{p0}+v_{i}$$

$$log \left( \mu_{i} \right)= \theta_{i}= \alpha_{\mu0}+E_{it}+\sum_{j=1}^{J} \text{β}\text{j}\text{. X}\text{it}\text{j}+ v_{i}+T_{t}$$

Model 23 (Poisson) and Model 63 (Negative binomial): the mean incidence with BYM random effect and unstructured temporal effect

$$logit {(p}_{i})=\log\left( \frac{p_{i}}{1-p_{i}} \right)=\alpha_{p0}+v_{i}$$

$$log \left( \mu_{i} \right)= \theta_{i}= \alpha_{\mu0}+E_{it}+\sum_{j=1}^{J} \text{β}\text{j}\text{. X}\text{it}\text{j}+ u_{i}+v_{i}+T_{t}$$

Model 24 (Poisson) and Model 64 (Negative binomial): the mean incidence with BYM random effect with spatial-temporal interaction term Type 1, and unstructured temporal effect

$$logit {(p}_{i})=\log\left( \frac{p_{i}}{1-p_{i}} \right)=\alpha_{p0}+v_{i}$$

$$log \left( \mu_{i} \right)= \theta_{i}= \alpha_{\mu0}+E_{it}+\sum_{j=1}^{J} \text{β}\text{j}\text{. X}\text{it}\text{j}+ u_{i}+v_{i}+T_{t}+\delta_{it}$$

In the third condition, the probability of zeroswas assumed to be constant across space and vary over time. So, it was assumed that $p_{it}=p_{t}$for all locations. Here, we used IID unstructured random effect $v_{t}$ for zero-probability that is change over time. Therefore, variation of the model can be developed as follow:

Model 25 (Poisson) and Model 65 (Negative binomial): the mean incidence with structured random effect and random walk of order 1 temporal effect

$$logit {(p}_{t})=\log\left( \frac{p_{t}}{1-p_{t}} \right)=\alpha_{p0}+v_{t}$$

$$log \left( \mu_{t} \right)= \theta_{i}= \alpha_{\mu0}+E_{it}+\sum_{j=1}^{J} \text{β}\text{j}\text{. X}\text{it}\text{j}+ u_{i}+T_{t}^{RW1}$$

Model 26 (Poisson) and Model 66 (Negative binomial): the mean incidence with unstructured random effect and random walk of order 1 temporal effect

$$logit {(p}_{t})=\log\left( \frac{p_{t}}{1-p_{t}} \right)=\alpha_{p0}+v_{t}$$

$$log \left( \mu_{t} \right)= \theta_{i}= \alpha_{\mu0}+E_{it}+\sum_{j=1}^{J} \text{β}\text{j}\text{. X}\text{it}\text{j}+ v_{i}+T_{t}^{RW1}$$

Model 27 (Poisson) and Model 67 (Negative binomial): the mean incidence with BYM random effect and random walk of order 1 temporal effect

$$logit {(p}_{t})=\log\left( \frac{p_{t}}{1-p_{t}} \right)=\alpha_{p0}+v_{t}$$

$$log \left( \mu_{t} \right)= \theta_{i}= \alpha_{\mu0}+E_{it}+\sum_{j=1}^{J} \text{β}\text{j}\text{. X}\text{it}\text{j}+ u_{i}+v_{i}+T_{t}^{RW1}$$

Model 28 (Poisson) and Model 68 (Negative binomial): the mean incidence with BYM random effect with spatial-temporal interaction term Type 1, and random walk of order 1 temporal effect

$$logit {(p}_{t})=\log\left( \frac{p_{t}}{1-p_{t}} \right)=\alpha_{p0}+v_{t}$$

$$log \left( \mu_{t} \right)= \theta_{i}= \alpha_{\mu0}+E_{it}+\sum_{j=1}^{J} \text{β}\text{j}\text{. X}\text{it}\text{j}+ u_{i}+v_{i}+T_{t}^{RW1}+\delta_{it}$$

Model 29 (Poisson) and Model 69 (Negative binomial): the mean incidence with structured random effect and unstructured temporal effect

$$logit {(p}_{t})=\log\left( \frac{p_{t}}{1-p_{t}} \right)=\alpha_{p0}+v_{t}$$

$$log \left( \mu_{t} \right)= \theta_{i}= \alpha_{\mu0}+E_{it}+\sum_{j=1}^{J} \text{β}\text{j}\text{. X}\text{it}\text{j}+ u_{i}+T_{t}$$

Model 30 (Poisson) and Model 70 (Negative binomial): the mean incidence with unstructured random effect and unstructured temporal effect

$$logit {(p}_{t})=\log\left( \frac{p_{t}}{1-p_{t}} \right)=\alpha_{p0}+v_{t}$$

$$log \left( \mu_{t} \right)= \theta_{i}= \alpha_{\mu0}+E_{it}+\sum_{j=1}^{J} \text{β}\text{j}\text{. X}\text{it}\text{j}+ v_{i}+T_{t}$$

Model 31 (Poisson) and Model 71 (Negative binomial): the mean incidence with BYM random effect and unstructured temporal effect

$$logit {(p}_{t})=\log\left( \frac{p_{t}}{1-p_{t}} \right)=\alpha_{p0}+v_{t}$$

$$log \left( \mu_{t} \right)= \theta_{i}= \alpha_{\mu0}+E_{it}+\sum_{j=1}^{J} \text{β}\text{j}\text{. X}\text{it}\text{j}+ u_{i}+v_{i}+T_{t}$$

Model 32 (Poisson) and Model 72 (Negative binomial): the mean incidence with BYM random effect with spatial-temporal interaction term Type 1, and unstructured temporal effect

$$logit {(p}_{t})=\log\left( \frac{p_{t}}{1-p_{t}} \right)=\alpha_{p0}+v_{t}$$

$$log \left( \mu_{t} \right)= \theta_{i}= \alpha_{\mu0}+E_{it}+\sum_{j=1}^{J} \text{β}\text{j}\text{. X}\text{it}\text{j}+ u_{i}+v_{i}+T_{t}+\delta_{it}$$

In the fourth condition, the probability of zeros was assumed to be vary across space and over time, or $p_{it}$for all locations. The IID unstructured random effect $v_{it}$ was used to indicate that zero-probability is change over time and space. Therefore, variation of the model can be written as follow:

Model 33 (Poisson) and Model 73 (Negative binomial): the mean incidence with structured random effect and random walk of order 1 temporal effect

$$logit {(p}_{it})=\log\left( \frac{p_{it}}{1-p_{it}} \right)=\alpha_{p0}+v_{it}$$

$$log \left( \mu_{it} \right)= \theta_{i}= \alpha_{\mu0}+E_{it}+\sum_{j=1}^{J} \text{β}\text{j}\text{. X}\text{it}\text{j}+ u_{i}+T_{t}^{RW1}$$

Model 34 (Poisson) and Model 74 (Negative binomial): the mean incidence with unstructured random effect and random walk of order 1 temporal effect

$$logit {(p}_{it})=\log\left( \frac{p_{it}}{1-p_{it}} \right)=\alpha_{p0}+v_{it}$$

$$log \left( \mu_{it} \right)= \theta_{i}= \alpha_{\mu0}+E_{it}+\sum_{j=1}^{J} \text{β}\text{j}\text{. X}\text{it}\text{j}+ v_{i}+T_{t}^{RW1}$$

Model 35 (Poisson) and Model 75 (Negative binomial): the mean incidence with BYM random effect and random walk of order 1 temporal effect

$$logit {(p}_{it})=\log\left( \frac{p_{it}}{1-p_{it}} \right)=\alpha_{p0}+v_{it}$$

$$log \left( \mu_{it} \right)= \theta_{i}= \alpha_{\mu0}+E_{it}+\sum_{j=1}^{J} \text{β}\text{j}\text{. X}\text{it}\text{j}+ u_{i}+v_{i}+T_{t}^{RW1}$$

Model 36 (Poisson) and Model 76 (Negative binomial): the mean incidence with BYM random effect with spatial-temporal interaction term Type 1, and random walk of order 1 temporal effect

$$logit {(p}_{it})=\log\left( \frac{p_{it}}{1-p_{it}} \right)=\alpha_{p0}+v_{it}$$

$$log \left( \mu_{it} \right)= \theta_{i}= \alpha_{\mu0}+E_{it}+\sum_{j=1}^{J} \text{β}\text{j}\text{. X}\text{it}\text{j}+ u_{i}+v_{i}+T_{t}^{RW1}+\delta_{it}$$

Model 37 (Poisson) and Model 77 (Negative binomial): the mean incidence with structured random effect and unstructured temporal effect

$$logit {(p}_{it})=\log\left( \frac{p_{it}}{1-p_{it}} \right)=\alpha_{p0}+v_{it}$$

$$log \left( \mu_{it} \right)= \theta_{i}= \alpha_{\mu0}+E_{it}+\sum_{j=1}^{J} \text{β}\text{j}\text{. X}\text{it}\text{j}+ u_{i}+T_{t}$$

Model 38 (Poisson) and Model 78 (Negative binomial): the mean incidence with unstructured random effect and unstructured temporal effect

$$logit {(p}_{it})=\log\left( \frac{p_{it}}{1-p_{it}} \right)=\alpha_{p0}+v_{it}$$

$$log \left( \mu_{it} \right)= \theta_{i}= \alpha_{\mu0}+E_{it}+\sum_{j=1}^{J} \text{β}\text{j}\text{. X}\text{it}\text{j}+ v_{i}+T_{t}$$

Model 39 (Poisson) and Model 79 (Negative binomial): the mean incidence with BYM random effect and unstructured temporal effect

$$logit {(p}_{it})=\log\left( \frac{p_{it}}{1-p_{it}} \right)=\alpha_{p0}+v_{it}$$

$$log \left( \mu_{it} \right)= \theta_{i}= \alpha_{\mu0}+E_{it}+\sum_{j=1}^{J} \text{β}\text{j}\text{. X}\text{it}\text{j}+ u_{i}+v_{i}+T_{t}$$

Model 40 (Poisson) and Model 80 (Negative binomial): the mean incidence with BYM random effect with spatial-temporal interaction term Type 1, and unstructured temporal effect

$$logit {(p}_{it})=\log\left( \frac{p_{it}}{1-p_{it}} \right)=\alpha_{p0}+v_{it}$$

$$log \left( \mu_{it} \right)= \theta_{i}= \alpha_{\mu0}+E_{it}+\sum_{j=1}^{J} \text{β}\text{j}\text{. X}\text{it}\text{j}+ u_{i}+v_{i}+T_{t}+\delta_{it}$$

The 80 models used in this analysis were systematically developed across various model types and assumptions. The first two sets of models, Regular Poisson and Regular Negative Binomial, each included 8 specifications without zero-inflation. These models served as baseline structures for comparing the influence of zero-inflation adjustments.

The next two sets of models incorporated zero-inflation to account for the excess zeros in the data. Thirty-two ZIP models were developed, each with varying assumptions about the zero-inflation component. These assumptions included constant zero-inflation across space and time, zero-inflation varying by space, varying by time, and varying across both space and time. Additionally, various spatiotemporal random effect structures were integrated into the models to capture the potential correlations across space and time.

Similarly, thirty-two ZINB models were developed. These models mirrored the ZIP models in terms of zero-inflation assumptions and spatiotemporal random effects but utilized the Negative Binomial distribution to better handle overdispersion in the data.

This comprehensive approach enabled a thorough evaluation of the impact of different statistical assumptions and model structures on the association between risk factors and DR-TB incidence. By comparing these models, we aimed to identify the most appropriate specifications that best represent the data-generating processes. This process allowed for robust insights into the factors contributing to DR-TB incidence. The evaluation metrics and estimated regression coefficients from these models are detailed further in Supplementary Documents S2 and S3.

**Glossary**

| *i* | District |
| --- | --- |
| *t* | Year |
| $SIR_{it}$ | Standardized Incidence Rate of DR-TB cases in *i* district and *t* year |
| $y_{it}$ | Number of notified DR-TB cases in *i* district and *t* year |
| $E_{it}$ | Number of expected DR-TB cases in *i* district and *t* year estimated from the population of reference $N_{it}$ |
| $N_{it}$ | Number of TB cases in *i* district and *t* year as the population of reference |
| $\mu_{it}$ | Mean incidence of notified DR-TB cases in *i* district and *t* year |
| $\theta_{it}$ | Log relative risk of DR-TB cases in *i* district and *t* year |
| *j* | Covariate |
| $\text{β}\text{0}$ | Overal intercept |
| $\text{X}\text{it}\text{j}$ | Value of *j* covariate in *i* district and *t* year |
| $\text{β}\text{j}$ | Value of regression coefficient associated with covariate $X\text{it}\text{j}$ |
| $u_{i}$ | Spatially structured random effect in *i* district |
| $v_{i}$ | Spatially unstructured random effect in *i* district |
| $T_{t}$ | Temporal random effect in *t* year |
| $\delta_{it}$ | Spatiotemporal interaction effect in *i* district and *t* year |
| $Y_{it}$ | A random variable representing the DR-TB case count in *i* district and *t* year; used in the formulation of statistical models. |
| *II* | Indicator function; represents the point mass at zero in the ZIP model |
| $p$ | Probability of a true DR-TB case (indicating the likelihood of being at risk for DR-TB) |
| $f$ | Base probability distribution for positive outcomes |
| *$\Omega_{i}$* | First-order spatial neighbours |
| *$n_{\delta_{i}}$* | Cardinality |
| *$\bar{u}_{\delta_{i}}$* | Average of the neighbourhood of the *i* district respectively |
| $T_{t-1}$ | Temporal random effect in the preceding *t* year |
| $\sigma_{u}^{2}$ | Temporal variance parameter |
| $\otimes$ | Kronecker product |
| $I$ | Identity matrix |
| $R_{\delta}$ | Kronecker product structure matrix; encodes the joint spatial and temporal dependency structure for the $\delta_{it}$ |
| $R_{v}$ | Spatial structure matrix; represents the spatial dependency |
| $R_{T}$ | Temporal structure matrix; defines the correlation across time points |
| $Pr$ | Likelihood to denote exceedance probability estimation |
| $q$ | Relative risk ratio |
| $\alpha$ | level of significant threshold ($0.05)$ |

**Supplementary document S4:** **Model evaluation metrics for analyzing the association between DR-TB incidence and risk factors**

This part provides a comprehensive comparison of model specifications for drug-resistant tuberculosis (DR-TB) incidence data, utilizing both Poisson and Negative Binomial likelihood functions. Table S2.1 presents the goodness-of-fit metrics, including the Deviance Information Criterion (DIC), Watanabe-Akaike Information Criterion (WAIC), and their respective effective number of parameters ($p$DIC and $p$WAIC). These metrics facilitate the evaluation of model performance and complexity across various configurations, aiding in the selection of the most appropriate model for analyzing DR-TB incidence.

| **Likelihood assumption** | **Model** | **Specification** | **DIC** | $\boldsymbol{p}$**DIC** | **WAIC** | $\boldsymbol{p}$**WAIC** |
| --- | --- | --- | --- | --- | --- | --- |
| Poisson | 1 | $u+ T_{t}^{RW1}$ | -9.53x10^293^ | -9.53x10^293^ | 1141585.46 | 536049 |
|  | 2 | $v+ T_{t}^{RW1}$ | 19867.43 | -1355.31 | 37286.87 | 8156.86 |
|  | 3 | $u+v+ T_{t}^{RW1}$ | 19894.85 | -1338.76 | 37030.36 | 8027.89 |
|  | 4 | $u+v+ T_{t}^{RW1}+ \delta_{it}$ | 14515.08 | 2008.15 | 14460.61 | 1412.55 |
|  | 5 | $u+ T_{t}$ | -4.46x10^294^ | -4.46x10^294^ | 560030.13 | 244309.1 |
|  | 6 | $v+ T_{t}$ | 19861.77 | -1358.20 | 37327.68 | 8177.75 |
|  | 7 | $u+v+ T_{t}$ | 19862.51 | -1355.03 | 37112.00 | 8069.08 |
|  | 8 | $u+v+ T_{t}+ \delta_{it}$ | 14513.68 | 2006.17 | 14461.57 | 1412.84 |
| Zero Inflated Poisson (constant over space and time) | 9 | $u+ T_{t}^{RW1}$ | N/A | N/A | N/A | N/A |
|  | 10 | $v+ T_{t}^{RW1}$ | 18952.32 | -913.3986 | 31382.10 | 5892.095 |
|  | 11 | $u+v+ T_{t}^{RW1}$ | 18956.98 | -908.1729 | 31396.89 | 5901.501 |
|  | 12 | $u+v+ T_{t}^{RW1}+ \delta_{it}$ | N/A | N/A | N/A | N/A |
|  | 13 | $u+ T_{t}$ | N/A | N/A | N/A | N/A |
|  | 14 | $v+ T_{t}$ | 18953.40 | -912.737 | 31371.21 | 5886.844 |
|  | 15 | $u+v+ T_{t}$ | 18957.75 | -907.7863 | 31379.87 | 5893.22 |
|  | 16 | $u+v+ T_{t}+ \delta_{it}$ | N/A | N/A | N/A | N/A |
| Zero Inflated Poisson (vary across space) | 17 | $u+ T_{t}^{RW1}$ | -5.75x10^294^ | -5.75x10^294^ | 566253.83 | 209051.2 |
|  | 18 | $v+ T_{t}^{RW1}$ | 22964.80 | -1013.06 | 40107.49 | 8310.67 |
|  | 19 | $u+v+ T_{t}^{RW1}$ | 23012.56 | -990.40 | 40086.81 | 8292.06 |
|  | 20 | $u+v+ T_{t}^{RW1}+ \delta_{it}$ | 17629.55 | 2351.10 | 17527.75 | 1679.8 |
|  | 21 | $u+ T_{t}$ | -6.24x10^294^ | -6.24x10^294^ | 485523.32 | 192415.2 |
|  | 22 | $v+ T_{t}$ | 22933.20 | -1028.89 | 40182.72 | 8348.85 |
|  | 23 | $u+v+ T_{t}$ | 22976.75 | -1007.45 | 40201.43 | 8350.14 |
|  | 24 | $u+v+ T_{t}+ \delta_{it}$ | 17627.50 | 2356.34 | 17523.36 | 1681.87 |
| Zero Inflated Poisson (vary over time) | 25 | $u+ T_{t}^{RW1}$ | -4.25x10^294^ | -4.25x10^294^ | 638952.63 | 281303.3 |
|  | 26 | $v+ T_{t}^{RW1}$ | 23038.45 | -1345.67 | 40204.86 | 8039.69 |
|  | 27 | $u+v+ T_{t}^{RW1}$ | 23060.64 | -1331.35 | 40186.12 | 8030.35 |
|  | 28 | $u+v+ T_{t}^{RW1}+ \delta_{it}$ | 17678.61 | 2011.50 | 17625.86 | 1418.08 |
|  | 29 | $u+ T_{t}$ | -6.25x10^294^ | -6.25x10^294^ | 573368.69 | 249753.9 |
|  | 30 | $v+ T_{t}$ | 23006.36 | -1361.69 | 40305.29 | 8090.55 |
|  | 31 | $u+v+ T_{t}$ | 23022.63 | -1350.44 | 40288.51 | 8081.98 |
|  | 32 | $u+v+ T_{t}+ \delta_{it}$ | 17677.84 | 2014.18 | 17623.80 | 1418.81 |
| Zero Inflated Poisson (vary across space and time) | 33 | $u+ T_{t}^{RW1}$ | -2.36x10^291^ | -2.36x10^291^ | 8580706.35 | 4246433 |
|  | 34 | $v+ T_{t}^{RW1}$ | 23545.53 | -1350.08 | 40752.58 | 8055.93 |
|  | 35 | $u+v+ T_{t}^{RW1}$ | 23550.66 | -1343.35 | 40710.54 | 8035.32 |
|  | 36 | $u+v+ T_{t}^{RW1}+ \delta_{it}$ | 18181.12 | 2023.88 | 18122.40 | 1419.21 |
|  | 37 | $u+ T_{t}$ | -3.99x10^291^ | -3.99x10^291^ | 1382235.12 | 653758.4 |
|  | 38 | $v+ T_{t}$ | 23508.04 | -1368.35 | 40831.32 | 8096.19 |
|  | 39 | $u+v+ T_{t}$ | 23530.48 | -1354.8 | 40796.87 | 8077.92 |
|  | 40 | $u+v+ T_{t}+ \delta_{it}$ | 18185.34 | 2011.53 | 18134.75 | 1421.30 |
| Negative Binomial | 41 | $u+ T_{t}^{RW1}$ | 16890.49 | 331.13 | 16999.36 | 354.28 |
|  | 42 | $v+ T_{t}^{RW1}$ | 16735.78 | 379.48 | 16827.70 | 387.36 |
|  | 43 | $u+v+ T_{t}^{RW1}$ | 16740.53 | 382.98 | 16827.17 | 387.01 |
|  | 44 | $u+v+ T_{t}^{RW1}+ \delta_{it}$ | 16659.68 | 659.73 | 16705.48 | 581.04 |
|  | 45 | $u+ T_{t}$ | 16890.02 | 331.18 | 16998.89 | 354.37 |
|  | 46 | $v+ T_{t}$ | 16735.69 | 380.56 | 16827.34 | 388.02 |
|  | 47 | $u+v+ T_{t}$ | 16739.65 | 383.56 | 16827.63 | 388.20 |
|  | 48 | $u+v+ T_{t}+ \delta_{it}$ | 16556.10 | 893.06 | 16587.82 | 744.18 |
| Zero Inflated Negative Binomial (constant over space and time) | 49 | $u+ T_{t}^{RW1}$ | 16828.26 | 346.85 | 16938.16 | 373.46 |
|  | 50 | $v+ T_{t}^{RW1}$ | 16648.93 | 387.45 | 16722.27 | 388.50 |
|  | 51 | $u+v+ T_{t}^{RW1}$ | 16646.22 | 388.62 | 16720.11 | 390.29 |
|  | 52 | $u+v+ T_{t}^{RW1}+ \delta_{it}$ | 16270.67 | 1039.12 | 16285.20 | 836.23 |
|  | 53 | $u+ T_{t}$ | 16826.24 | 348.73 | 16934.62 | 374.24 |
|  | 54 | $v+ T_{t}$ | 16649.52 | 391.28 | 16722.09 | 391.07 |
|  | 55 | $u+v+ T_{t}$ | 16645.73 | 389.62 | 16718.91 | 390.63 |
|  | 56 | $u+v+ T_{t}+ \delta_{it}$ | 16655.92 | 391.14 | 16719.17 | 385.54 |
| Zero Inflated Negative Binomial (vary across space) | 57 | $u+ T_{t}^{RW1}$ | 19980.52 | 669.26 | 20044.64 | 620.08 |
|  | 58 | $v+ T_{t}^{RW1}$ | 19826.12 | 719.60 | 19873.74 | 654.82 |
|  | 59 | $u+v+ T_{t}^{RW1}$ | 19823.95 | 719.55 | 19877.37 | 655.57 |
|  | 60 | $u+v+ T_{t}^{RW1}+ \delta_{it}$ | 19643.03 | 1186.12 | 19651.73 | 989.82 |
|  | 61 | $u+ T_{t}$ | 19979.67 | 670.41 | 20044.29 | 621.31 |
|  | 62 | $v+ T_{t}$ | 19826.13 | 721.06 | 19873.49 | 655.74 |
|  | 63 | $u+v+ T_{t}$ | 19822.20 | 721.01 | 19876.00 | 657.37 |
|  | 64 | $u+v+ T_{t}+ \delta_{it}$ | 19746.29 | 988.88 | 19755.74 | 842.21 |
| Zero Inflated Negative Binomial (vary over time) | 65 | $u+ T_{t}^{RW1}$ | 20054.11 | 336.77 | 20162.24 | 359.56 |
|  | 66 | $v+ T_{t}^{RW1}$ | 19899.13 | 386.06 | 19990.82 | 393.68 |
|  | 67 | $u+v+ T_{t}^{RW1}$ | 19896.09 | 386.54 | 19994.14 | 395.29 |
|  | 68 | $u+v+ T_{t}^{RW1}+ \delta_{it}$ | 19735.63 | 795.89 | 19797.68 | 694.30 |
|  | 69 | $u+ T_{t}$ | 20053.66 | 339.17 | 20161.30 | 361.08 |
|  | 70 | $v+ T_{t}$ | 19898.36 | 383.77 | 19990.85 | 392.46 |
|  | 71 | $u+v+ T_{t}$ | 19896.55 | 389.86 | 19992.81 | 396.86 |
|  | 72 | $u+v+ T_{t}+ \delta_{it}$ | 19732.71 | 833.09 | 19780.60 | 710.12 |
| Zero Inflated Negative Binomial (vary across space and time) | 73 | $u+ T_{t}^{RW1}$ | 20559.80 | 331.61 | 20669.26 | 355.41 |
|  | 74 | $v+ T_{t}^{RW1}$ | 20412.15 | 386.87 | 20498.92 | 389.48 |
|  | 75 | $u+v+ T_{t}^{RW1}$ | 20395.56 | 379.89 | 20498.56 | 392.82 |
|  | 76 | $u+v+ T_{t}^{RW1}+ \delta_{it}$ | 20204.02 | 752.95 | 20306.01 | 691.17 |
|  | 77 | $u+ T_{t}$ | 20561.12 | 333.83 | 20669.10 | 355.97 |
|  | 78 | $v+ T_{t}$ | 20407.84 | 375.44 | 20503.48 | 386.94 |
|  | 79 | $u+v+ T_{t}$ | 20404.05 | 383.72 | 20500.64 | 391.05 |
|  | 80 | $u+v+ T_{t}+ \delta_{it}$ | 20196.60 | 887.09 | 20246.15 | 747.50 |

**Table S4.1** The goodness-of-fit metrics for various model specifications under different likelihood assumptions, including Poisson and Negative Binomial distributions.

**
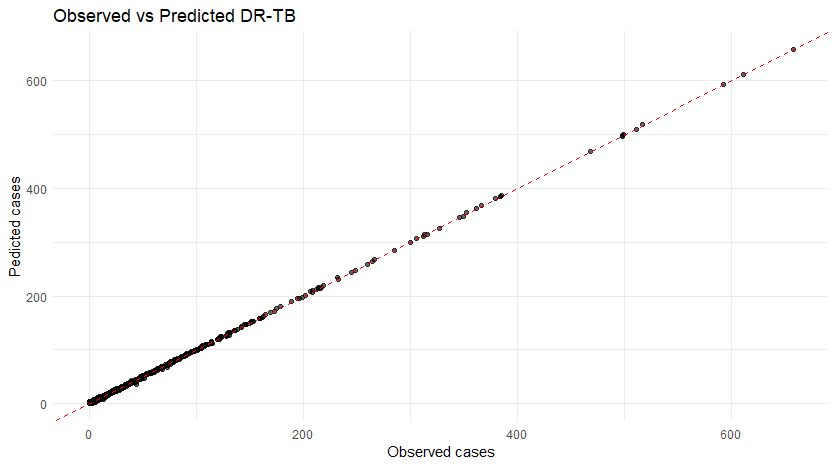
**

**Figure S4.1** Scatter plot comparing observed outcomes with predicted values from the best-fitting model (Poisson with RW1 temporal effect, BYM spatial structure, and type 1 spatiotemporal interaction)

**Supplementary document S5: Estimated regression coefficients of risk factors under likelihood assumptions and model specifications of DR-TB incidence**

This section presents the estimated regression coefficients for various risk factors associated with drug-resistant tuberculosis (DR-TB) incidence, as determined across multiple model specifications. The analysis encompasses both Poisson and Negative Binomial likelihood functions, with and without zero-inflation components, to account for overdispersion and excess zeros in the data. The estimated coefficients provide insights into the strength and direction of the association between each risk factor and DR-TB incidence, facilitating a comprehensive understanding of the determinants influencing DR-TB occurrence.

| **Model** | **Coefficients, posterior mean (95% CrI)** | | | | | | | |
| --- | --- | --- | --- | --- | --- | --- | --- | --- |
|  | **Treatment coverage** | **Treatment complete rate** | **Treatment success rate** | **Health center per 100,000 population** | **Proportion of insured population** | **Proportion of population below poverty** | **Proportion off households with access to sanitation** | **MHDI** |
| 1 | -0.569 (-0.609 to -0.529) | 0.492 (0.382 to 0.601) | -0.263 (-0.397 to -0.130) | -0.119 (-0.128 to -0.110) | 0.008 (0.007 to 0.008) | 0.102 (0.094 to 0.109) | 0.036 (0.034 to 0.038) | 0.024 (0.011 to 0.036) |
| 2 | -0.228 (-0.269 to -0.187) | 0.984 (0.871 to 1.096) | -0.852 (-0.991 to -0.714) | -0.021 (-0.034 to -0.007) | 0.001 (0.001 to 0.002) | -0.003 (-0.014 to 0.008) | 0.019 (0.017 to 0.021) | 0.022 (0.007 to 0.036) |
| 3 | -0.229 (-0.270 to -0.188) | 0.976 (0.863 to 1.088) | -0.842 (-0.981 to -0.704) | -0.020 (-0.035 to -0.006) | 0.001 (0.001 to 0.002) | -0.003 (-0.015 to 0.008) | 0.019 (0.017 to 0.021) | 0.020 (0.005 to 0.035) |
| 4 | -0.117 (-0.267 to 0.033) | 0.852 (0.576 to 1.128) | -0.667 (-0.989 to -0.345) | -0.014 (-0.030 to 0.003) | 0.001 (0.000 to 0.003) | 0.027 (0.013 to 0.041) | 0.007 (0.003 to 0.011) | 0.066 (0.048 to 0.084) |
| 5 | -0.544 (-0.610 to -0.352) | 0.528 (0.359 to 1.087) | -0.313 (-0.977 to -0.132) | -0.119 (-0.128 to -0.110) | 0.002 (0.001 to 0.008) | 0.101 (0.094 to 0.109) | 0.036 (0.034 to 0.038) | 0.023 (0.011 to 0.035) |
| 6 | -0.229 (-0.270 to -0.188) | 0.985 (0.873 to 1.097) | -0.852 (-0.991 to -0.714) | -0.021 (-0.034 to -0.008) | 0.001 (0.001 to 0.002) | -0.001 (-0.012 to 0.010) | 0.019 (0.017 to 0.021) | 0.021 (0.007 to 0.036) |
| 7 | -0.231 (-0.272 to -0.190) | 0.978 (0.865 to 1.090) | -0.843 (-0.981 to -0.704) | -0.021 (-0.035 to -0.007) | 0.001 (0.001 to 0.002) | -0.002 (-0.013 to 0.010) | 0.019 (0.017 to 0.021) | 0.020 (0.005 to 0.035) |
| 8 | -0.119 (-0.269 to 0.031) | 0.890 (0.612 to 1.169) | -0.676 (-0.998 to -0.354) | -0.014 (-0.030 to 0.003) | 0.001 (0.000 to 0.003) | 0.028 (0.014 to 0.041) | 0.007 (0.003 to 0.011) | 0.066 (0.048 to 0.084) |
| 9 | N/A | N/A | N/A | N/A | N/A | N/A | N/A | N/A |
| 10 | -0.259 (-0.300 to -0.218) | 0.651 (0.535 to 0.768) | -0.652 (-0.797 to -0.507) | -0.007 (-0.020 to 0.006) | 0.001 (0.001 to 0.002) | 0.004 (-0.007 to 0.014) | 0.014 (0.012 to 0.016) | 0.031 (0.018 to 0.045) |
| 11 | -0.261 (-0.302 to -0.220) | 0.646 (0.530 to 0.762) | -0.639 (-0.784 to -0.494) | -0.006 (-0.020 to 0.008) | 0.001 (0.001 to 0.002) | 0.004 (-0.008 to 0.015) | 0.014 (0.012 to 0.016) | 0.031 (0.017 to 0.045) |
| 12 | N/A | N/A | N/A | N/A | N/A | N/A | N/A | N/A |
| 13 | N/A | N/A | N/A | N/A | N/A | N/A | N/A | N/A |
| 14 | -0.260 (-0.301 to -0.220) | 0.653 (0.537 to 0.769) | -0.653 (-0.798 to -0.508) | -0.007 (-0.020 to 0.006) | 0.001 (0.001 to 0.002) | 0.005 (-0.006 to 0.015) | 0.014 (0.012 to 0.016) | 0.032 (0.018 to 0.045) |
| 15 | -0.263 (-0.304 to -0.222) | 0.649 (0.533 to 0.765) | -0.641 (-0.785 to -0.496) | -0.006 (-0.020 to 0.007) | 0.001 (0.001 to 0.002) | 0.005 (-0.006 to 0.016) | 0.014 (0.012 to 0.016) | 0.032 (0.018 to 0.046) |
| 16 | N/A | N/A | N/A | N/A | N/A | N/A | N/A | N/A |
| 17 | -0.569 (-0.609 to -0.529) | 0.492 (0.383 to 0.601) | -0.263 (-0.396 to -0.129) | -0.119 (-0.128 to -0.110) | 0.008 (0.007 to 0.008) | 0.102 (0.094 to 0.109) | 0.036 (0.034 to 0.038) | 0.024 (0.012 to 0.036) |
| 18 | -0.228 (-0.269 to -0.187) | 0.984 (0.872 to 1.096) | -0.852 (-0.990 to -0.713) | -0.021 (-0.034 to -0.008) | 0.001 (0.001 to 0.002) | -0.001 (-0.012 to 0.010) | 0.019 (0.017 to 0.021) | 0.023 (0.008 to 0.037) |
| 19 | -0.229 (-0.270 to -0.188) | 0.975 (0.863 to 1.088) | -0.841 (-0.980 to -0.703) | -0.021 (-0.035 to -0.006) | 0.001 (0.001 to 0.002) | -0.001 (-0.013 to 0.010) | 0.019 (0.017 to 0.021) | 0.021 (0.006 to 0.036) |
| 20 | -0.121 (-0.272 to 0.029) | 0.853 (0.577 to 1.129) | -0.667 (-0.988 to -0.345) | -0.013 (-0.030 to 0.003) | 0.001 (0.000 to 0.003) | 0.028 (0.015 to 0.042) | 0.007 (0.003 to 0.011) | 0.067 (0.049 to 0.085) |
| 21 | -0.449 (-0.602 to -0.335) | 0.587 (-0.112 to 1.135) | -0.307 (-0.965 to -0.132) | -0.119 (-0.129 to -0.110) | 0.007 (0.001 to 0.008) | 0.102 (0.094 to 0.109) | 0.025 (0.017 to 0.037) | 0.023 (0.011 to 0.036) |
| 22 | -0.229 (-0.270 to -0.188) | 0.985 (0.873 to 1.097) | -0.852 (-0.991 to -0.714) | -0.021 (-0.034 to -0.008) | 0.001 (0.001 to 0.002) | -0.001 (-0.012 to 0.010) | 0.019 (0.017 to 0.021) | 0.022 (0.007 to 0.036) |
| 23 | -0.231 (-0.272 to -0.190) | 0.979 (0.866 to 1.091) | -0.843 (-0.982 to -0.705) | -0.021 (-0.035 to -0.007) | 0.001 (0.001 to 0.002) | -0.002 (-0.013 to 0.010) | 0.019 (0.017 to 0.021) | 0.020 (0.005 to 0.035) |
| 24 | -0.116 (-0.266 to 0.032) | 0.873 (0.596 to 1.151) | -0.669 (-0.991 to -0.346) | -0.014 (-0.030 to 0.003) | 0.001 (0.000 to 0.003) | 0.028 (0.014 to 0.041) | 0.007 (0.003 to 0.011) | 0.066 (0.049 to 0.084) |
| 25 | -0.569 (-0.609 to -0.529) | 0.492 (0.382 to 0.601) | -0.263 (-0.396 to -0.129) | -0.116 (-0.128 to -0.074) | 0.008 (0.004 to 0.008) | 0.099 (0.063 to 0.109) | 0.036 (0.034 to 0.038) | 0.020 (-0.038 to 0.036) |
| 26 | -0.228 (-0.269 to -0.187) | 0.983 (0.871 to 1.096) | -0.851 (-0.990 to -0.713) | -0.021 (-0.034 to -0.008) | 0.001 (0.001 to 0.002) | -0.001 (-0.012 to 0.010) | 0.019 (0.017 to 0.021) | 0.023 (0.008 to 0.037) |
| 27 | -0.229 (-0.270 to -0.188) | 0.976 (0.864 to 1.088) | -0.842 (-0.980 to -0.703) | -0.021 (-0.035 to -0.006) | 0.001 (0.001 to 0.002) | -0.001 (-0.013 to 0.010) | 0.019 (0.017 to 0.021) | 0.021 (0.006 to 0.036) |
| 28 | -0.114 (-0.263 to 0.034) | 0.848 (0.574 to 1.123) | -0.666 (-0.987 to -0.345) | -0.014 (-0.030 to 0.003) | 0.001 (0.000 to 0.003) | 0.028 (0.014 to 0.041) | 0.007 (0.003 to 0.011) | 0.067 (0.049 to 0.084) |
| 29 | -0.461 (-0.603 to -0.336) | 0.579 (-0.076 to 1.124) | -0.357 (-1.011 to -0.134) | -0.115 (-0.129 to -0.033) | 0.006 (0.001 to 0.008) | 0.100 (0.063 to 0.109) | 0.031 (0.018 to 0.037) | 0.023 (0.011 to 0.036) |
| 30 | -0.229 (-0.270 to -0.188) | 0.985 (0.872 to 1.097) | -0.852 (-0.991 to -0.714) | -0.021 (-0.034 to -0.008) | 0.001 (0.001 to 0.002) | -0.001 (-0.012 to 0.010) | 0.019 (0.017 to 0.021) | 0.022 (0.007 to 0.036) |
| 31 | -0.231 (-0.272 to -0.190) | 0.978 (0.865 to 1.090) | -0.843 (-0.981 to -0.704) | -0.021 (-0.035 to -0.007) | 0.001 (0.001 to 0.002) | -0.002 (-0.013 to 0.010) | 0.019 (0.017 to 0.021) | 0.020 (0.005 to 0.035) |
| 32 | -0.118 (-0.269 to 0.032) | 0.889 (0.611 to 1.168) | -0.676 (-0.999 to -0.354) | -0.014 (-0.030 to 0.003) | 0.001 (0.001 to 0.003) | 0.028 (0.014 to 0.041) | 0.007 (0.003 to 0.011) | 0.066 (0.048 to 0.084) |
| 33 | -0.569 (-0.609 to -0.529) | 0.491 (0.382 to 0.600) | -0.262 (-0.396 to -0.129) | -0.119 (-0.129 to -0.110) | 0.008 (0.007 to 0.008) | 0.102 (0.094 to 0.109) | 0.036 (0.034 to 0.038) | -0.092 (-0.111 to -0.035) |
| 34 | -0.228 (-0.269 to -0.187) | 0.982 (0.869 to 1.094) | -0.850 (-0.989 to -0.712) | -0.021 (-0.034 to -0.008) | 0.001 (0.001 to 0.002) | -0.001 (-0.013 to 0.010) | 0.019 (0.017 to 0.021) | 0.023 (0.008 to 0.037) |
| 35 | -0.230 (-0.271 to -0.189) | 0.978 (0.866 to 1.091) | -0.843 (-0.981 to -0.704) | -0.021 (-0.035 to -0.007) | 0.001 (0.001 to 0.002) | -0.002 (-0.013 to 0.010) | 0.019 (0.017 to 0.021) | 0.021 (0.006 to 0.036) |
| 36 | -0.112 (-0.262 to 0.037) | 0.857 (0.578 to 1.135) | -0.666 (-0.992 to -0.341) | -0.015 (-0.031 to 0.002) | 0.001 (0.000 to 0.003) | 0.027 (0.013 to 0.041) | 0.007 (0.003 to 0.011) | 0.065 (0.047 to 0.083) |
| 37 | -0.514 (-0.608 to -0.343) | 0.650 (0.388 to 1.132) | -0.304 (-0.960 to -0.132) | -0.119 (-0.129 to -0.110) | 0.008 (0.007 to 0.008) | 0.102 (0.094 to 0.109) | 0.036 (0.034 to 0.038) | 0.012 (-0.046 to 0.035) |
| 38 | -0.229 (-0.270 to -0.188) | 0.984 (0.871 to 1.096) | -0.851 (-0.990 to -0.713) | -0.021 (-0.035 to -0.008) | 0.001 (0.001 to 0.002) | -0.002 (-0.013 to 0.009) | 0.019 (0.017 to 0.021) | 0.021 (0.007 to 0.036) |
| 39 | -0.231 (-0.272 to -0.190) | 0.977 (0.865 to 1.090) | -0.842 (-0.981 to -0.704) | -0.021 (-0.035 to -0.007) | 0.001 (0.001 to 0.002) | -0.002 (-0.013 to 0.010) | 0.019 (0.017 to 0.021) | 0.020 (0.005 to 0.035) |
| 40 | -0.122 (-0.271 to 0.027) | 0.893 (0.616 to 1.170) | -0.675 (-0.997 to -0.354) | -0.014 (-0.031 to 0.003) | 0.001 (0.000 to 0.003) | 0.027 (0.014 to 0.041) | 0.007 (0.003 to 0.011) | 0.065 (0.048 to 0.083) |
| 41 | -0.024 (-0.164 to 0.116) | 0.839 (0.573 to 1.105) | -0.704 (-1.012 to -0.397) | -0.013 (-0.025 to 0.000) | 0.001 (0.00 to 0.002) | 0.041 (0.030 to 0.051) | 0.006 (0.002 to 0.010) | 0.079 (0.062 to 0.096) |
| 42 | -0.142 (-0.289 to 0.003) | 0.848 (0.578 to 1.118) | -0.645 (-0.962 to -0.327) | -0.016 (-0.031 to -0.001) | 0.002 (0.000 to 0.003) | 0.028 (0.015 to 0.041) | 0.007 (0.003 to 0.011) | 0.068 (0.050 to 0.085) |
| 43 | -0.146 (-0.293 to 0.002) | 0.818 (0.547 to 1.089) | -0.592 (-0.911 to -0.272) | -0.010 (-0.026 to 0.007) | 0.002 (0.000 to 0.003) | 0.027 (0.013 to 0.041) | 0.006 (0.001 to 0.010) | 0.072 (0.054 to 0.091) |
| 44 | -0.126 (-0.276 to 0.022) | 0.836 (0.564 to 1.108) | -0.617 (-0.937 to -0.296) | -0.009 (-0.026 to 0.008) | 0.001 (0.000 to 0.003) | 0.027 (0.013 to 0.041) | 0.005 (0.001 to 0.010) | 0.072 (0.054 to 0.054) |
| 45 | -0.027 (-0.168 to 0.114) | 0.891 (0.621 to 1.162) | -0.718 (-1.026 to -0.410) | -0.012 (-0.025 to 0.000) | 0.001 (0.000 to 0.002) | 0.041 (0.031 to 0.051) | 0.007 (0.003 to 0.011) | 0.079 (0.062 to 0.096) |
| 46 | -0.144 (-0.292 to 0.003) | 0.898 (0.624 to 1.172) | -0.660 (-0.978 to -0.342) | -0.016 (-0.031 to -0.001) | 0.002 (0.000 to 0.003) | 0.028 (0.015 to 0.041) | 0.007 (0.003 to 0.011) | 0.068 (0.051 to 0.085) |
| 47 | -0.150 (-0.298 to -0.001) | 0.868 (0.593 to 1.143) | -0.607 (-0.926 to -0.287) | -0.010 (-0.026 to 0.007) | 0.002 (0.000 to 0.003) | 0.028 (0.014 to 0.042) | 0.006 (0.002 to 0.010) | 0.073 (0.054 to 0.091) |
| 48 | -0.112 (-0.262 to 0.038) | 0.900 (0.623 to 1.176) | -0.653 (-0.974 to -0.333) | -0.008 (-0.024 to 0.009) | 0.001 (0.000 to 0.003) | 0.027 (0.013 to 0.041) | 0.005 (0.001 to 0.010) | 0.073 (0.055 to 0.091) |
| 49 | -0.056 (-0.184 to 0.072) | 0.739 (0.491 to 0.988) | -0.608 (-0.905 to -0.311) | -0.008 (-0.020 to 0.004) | 0.001 (0.000 to 0.002) | 0.041 (0.031 to 0.051) | 0.006 (0.002 to 0.010) | 0.074 (0.058 to 0.090) |
| 50 | -0.161 (-0.296 to -0.027) | 0.726 (0.472 to 0.982) | -0.562 (-0.868 to -0.255) | -0.013 (-0.027 to 0.002) | 0.002 (0.000 to 0.003) | 0.027 (0.014 to 0.039) | 0.007 (0.003 to 0.011) | 0.064 (0.048 to 0.081) |
| 51 | -0.164 (-0.298 to -0.030) | 0.696 (0.442 to 0.951) | -0.500 (-0.807 to -0.193) | -0.010 (-0.026 to 0.006) | 0.002 (0.000 to 0.003) | 0.028 (0.015 to 0.041) | 0.006 (0.002 to 0.010) | 0.068 (0.050 to 0.085) |
| 52 | -0.164 (-0.298 to -0.030) | 0.699 (0.445 to 0.954) | -0.501 (-0.808 to -0.194) | -0.010 (-0.026 to 0.006) | 0.002 (0.000 to 0.003) | 0.028 (0.015 to 0.041) | 0.006 (0.002 to 0.010) | 0.067 (0.050 to 0.085) |
| 53 | -0.061 (-0.191 to 0.069) | 0.805 (0.550 to 1.058) | -0.627 (-0.925 to -0.330) | -0.008 (-0.020 to 0.004) | 0.001 (0.000 to 0.002) | 0.041 (0.031 to 0.051) | 0.006 (0.002 to 0.010) | 0.074 (0.058 to 0.090) |
| 54 | -0.167 (-0.302 to -0.032) | 0.784 (0.524 to 1.044) | -0.580 (-0.886 to -0.273) | -0.013 (-0.027 to 0.002) | 0.002 (0.000 to 0.003) | 0.027 (0.015 to 0.040) | 0.007 (0.003 to 0.011) | 0.064 (0.048 to 0.081) |
| 55 | -0.263 (-0.304 to -0.222) | 0.649 (0.533 to 0.766) | -0.641 (-0.785 to -0.496) | -0.006 (-0.020 to 0.007) | 0.001 (0.001 to 0.002) | 0.005 (-0.006 to 0.016) | 0.014 (0.012 to 0.016) | 0.032 (0.018 to 0.046) |
| 56 | -0.169 (-0.305 to -0.033) | 0.779 (0.519 to 1.037) | -0.530 (-0.839 to -0.222) | -0.010 (-0.026 to 0.006) | 0.002 (0.000 to 0.003) | 0.029 (0.016 to 0.043) | 0.007 (0.003 to 0.011) | 0.068 (0.051 to 0.086) |
| 57 | -0.025 (-0.165 to 0.115) | 0.839 (0.573 to 1.106) | -0.703 (-1.011 to -0.396) | -0.013 (-0.025 to 0.000) | 0.001 (0.000 to 0.002) | 0.041 (0.031 to 0.052) | 0.006 (0.002 to 0.010) | 0.079 (0.062 to 0.096) |
| 58 | -0.142 (-0.289 to 0.003) | 0.848 (0.578 to 1.118) | -0.643 (-0.960 to -0.325) | -0.016 (-0.031 to -0.001) | 0.002 (0.000 to 0.003) | 0.028 (0.015 to 0.041) | 0.006 (0.002 to 0.011) | 0.068 (0.051 to 0.086) |
| 59 | -0.144 (-0.292 to 0.003) | 0.820 (0.549 to 1.092) | -0.598 (-0.917 to -0.278) | -0.017 (-0.033 to 0.000) | 0.002 (0.000 to 0.003) | 0.030 (0.017 to 0.044) | 0.006 (0.002 to 0.010) | 0.072 (0.054 to 0.090) |
| 60 | -0.107 (-0.257 to 0.043) | 0.859 (0.587 to 1.133) | -0.650 (-0.970 to -0.330) | -0.019 (-0.035 to -0.002) | 0.001 (0.000 to 0.003) | 0.031 (0.017 to 0.044) | 0.006 (0.002 to 0.011) | 0.072 (0.054 to 0.090) |
| 61 | -0.027 (-0.169 to 0.114) | 0.893 (0.623 to 1.164) | -0.719 (-1.028 to -0.411) | -0.012 (-0.025 to 0.000) | 0.001 (0.000 to 0.002) | 0.041 (0.031 to 0.052) | 0.006 (0.002 to 0.010) | 0.079 (0.062 to 0.096) |
| 62 | -0.144 (-0.292 to 0.004) | 0.887 (0.614 to 1.161) | -0.655 (-0.973 to -0.336) | -0.016 (-0.031 to -0.001) | 0.002 (0.000 to 0.003) | 0.029 (0.016 to 0.042) | 0.006 (0.002 to 0.011) | 0.068 (0.051 to 0.086) |
| 63 | -0.148 (-0.297 to 0.001) | 0.001 (0.594 to 1.146) | -0.614 (-0.934 to -0.294) | -0.016 (-0.033 to 0.000) | 0.002 (0.000 to 0.003) | 0.031 (0.017 to 0.045) | 0.006 (0.002 to 0.011) | 0.072 (0.054 to 0.091) |
| 64 | -0.120 (-0.269 to 0.028) | 0.886 (0.611 to 1.161) | -0.644 (-0.964 to -0.324) | -0.018 (-0.034 to -0.001) | 0.001 (0.000 to 0.003) | 0.031 (0.018 to 0.045) | 0.006 (0.002 to 0.010) | 0.073 (0.055 to 0.091) |
| 65 | -0.024 (-0.164 to 0.116) | 0.839 (0.573 to 1.105) | -0.703 (-1.010 to -0.395) | -0.013 (-0.025 to 0.000) | 0.001 (0.000 to 0.002) | 0.041 (0.031 to 0.051) | 0.006 (0.002 to 0.010) | 0.079 (0.062 to 0.096) |
| 66 | -0.143 (-0.289 to 0.003) | 0.848 (0.578 to 1.118) | -0.643 (-0.961 to -0.326) | -0.016 (-0.031 to -0.001) | 0.002 (0.000 to 0.003) | 0.028 (0.015 to 0.041) | 0.007 (0.002 to 0.011) | 0.068 (0.051 to 0.086) |
| 67 | -0.144 (-0.291 to 0.003) | 0.820 (0.549 to 1.091) | -0.597 (-0.917 to -0.278) | -0.017 (-0.033 to 0.000) | 0.002 (0.000 to 0.003) | 0.030 (0.016 to 0.044) | 0.006 (0.002 to 0.011) | 0.072 (0.053 to 0.090) |
| 68 | -0.108 (-0.257 to 0.041) | 0.856 (0.582 to 1.131) | -0.644 (-0.965 to -0.324) | -0.019 (-0.035 to -0.002) | 0.001 (0.000 to 0.003) | 0.030 (0.017 to 0.044) | 0.006 (0.002 to 0.011) | 0.071 (0.053 to 0.089) |
| 69 | -0.027 (-0.168 to 0.114) | 0.891 (0.621 to 1.162) | -0.718 (-1.027 to -0.411) | -0.012 (-0.025 to 0.000) | 0.001 (0.000 to 0.002) | 0.041 (0.031 to 0.052) | 0.006 (0.002 to 0.010) | 0.079 (0.062 to 0.096) |
| 70 | -0.144 (-0.292 to 0.004) | 0.903 (0.628 to 1.178) | -0.662 (-0.981 to -0.344) | -0.016 (-0.031 to -0.001) | 0.002 (0.000 to 0.003) | 0.029 (0.015 to 0.042) | 0.007 (0.003 to 0.011) | 0.068 (0.051 to 0.085) |
| 71 | -0.149 (-0.298 to 0.000) | 0.876 (0.600 to 1.151) | -0.616 (-0.936 to -0.296) | -0.016 (-0.033 to 0.000) | 0.002 (0.000 to 0.003) | 0.031 (0.017 to 0.044) | 0.007 (0.002 to 0.011) | 0.072 (0.053 to 0.090) |
| 72 | -0.110 (-0.259 to 0.040) | 0.903 (0.627 to 1.179) | -0.661 (-0.982 to -0.341) | -0.019 (-0.035 to -0.002) | 0.001 (0.000 to 0.003) | 0.031 (0.017 to 0.044) | 0.007 (0.002 to 0.011) | 0.071 (0.053 to 0.090) |
| 73 | -0.024 (-0.165 to 0.116) | 0.839 (0.573 to 1.106) | -0.703 (-1.011 to -0.395) | -0.013 (-0.026 to 0.000) | 0.001 (0.000 to 0.002) | 0.041 (0.031 to 0.051) | 0.006 (0.002 to 0.010) | 0.079 (0.062 to 0.096) |
| 74 | -0.142 (-0.289 to 0.004) | 0.849 (0.579 to 1.120) | -0.643 (-0.961 to -0.325) | -0.016 (-0.031 to -0.001) | 0.002 (0.000 to 0.003) | 0.028 (0.015 to 0.041) | 0.007 (0.003 to 0.011) | 0.068 (0.051 to 0.085) |
| 75 | -0.143 (-0.286 to 0.001) | 0.815 (0.547 to 1.083) | -0.595 (-0.912 to -0.279) | -0.016 (-0.033 to 0.000) | 0.002 (0.000 to 0.003) | 0.030 (0.017 to 0.044) | 0.007 (0.002 to 0.011) | 0.072 (0.054 to 0.090) |
| 76 | -0.110 (-0.259 to 0.039) | 0.860 (0.584 to 1.136) | -0.645 (-0.968 to -0.323) | -0.019 (-0.036 to -0.002) | 0.002 (0.000 to 0.003) | 0.030 (0.017 to 0.044) | 0.007 (0.003 to 0.011) | 0.071 (0.053 to 0.089) |
| 77 | -0.027 (-0.168 to 0.114) | 0.890 (0.619 to 1.160) | -0.718 (-1.026 to -0.410) | -0.012 (-0.025 to 0.000) | 0.001 (0.000 to 0.002) | 0.041 (0.031 to 0.051) | 0.007 (0.003 to 0.011) | 0.079 (0.062 to 0.096) |
| 78 | -0.130 (-0.272 to 0.013) | 0.890 (0.621 to 1.159) | -0.662 (-0.976 to -0.349) | -0.015 (-0.030 to -0.001) | 0.002 (0.000 to 0.003) | 0.028 (0.016 to 0.041) | 0.007 (0.003 to 0.011) | 0.068 (0.051 to 0.085) |
| 79 | -0.146 (-0.295 to 0.002) | 0.880 (0.607 to 1.153) | -0.619 (-0.938 to -0.300) | -0.016 (-0.033 to 0.000) | 0.002 (0.000 to 0.003) | 0.031 (0.017 to 0.044) | 0.007 (0.003 to 0.011) | 0.072 (0.053 to 0.090) |
| 80 | -0.109 (-0.256 to 0.038) | 0.910 (0.637 to 1.184) | -0.666 (-0.986 to -0.347) | -0.019 (-0.036 to -0.003) | 0.002 (0.000 to 0.003) | 0.030 (0.017 to 0.044) | 0.007 (0.003 to 0.011) | 0.071 (0.053 to 0.088) |

**Table S5.1** Estimated regression coefficients for risk factors across various likelihood assumptions and model specifications of DR-TB incidence.


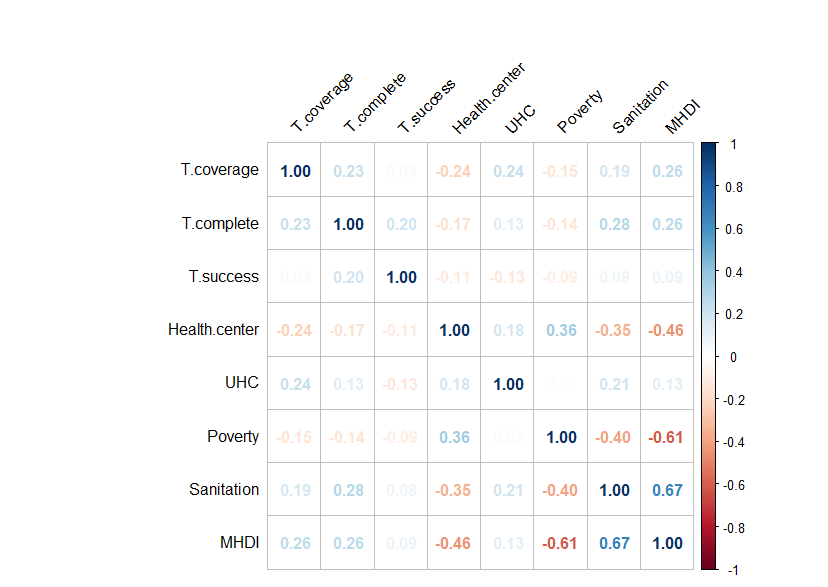


**Figure S5.1** Correlation matrix of covariates included in the analysis of drug-resistant tuberculosis.
